# Enrichment of G2/M cell cycle phase in human pluripotent stem cells enhances HDR-mediated gene repair with customizable endonucleases

**DOI:** 10.1038/srep21264

**Published:** 2016-02-18

**Authors:** Diane Yang, Marissa A Scavuzzo, Jolanta Chmielowiec, Robert Sharp, Aleksandar Bajic, Malgorzata Borowiak

**Affiliations:** 1 Molecular and Cellular Biology Department, Baylor College of Medicine, One Baylor Plaza, Houston, TX 77030, USA; 2 Program in Developmental Biology, Baylor College of Medicine, One Baylor Plaza, Houston, TX 77030, USA; 3 Stem Cells and Regenerative Medicine Center, Baylor College of Medicine, One Baylor Plaza, Houston, TX 77030, USA; 4 Center for Cell and Gene Therapy, Baylor College of Medicine, Texas Children’s Hospital and Houston Methodist Hospital, Houston, TX 77030, USA; 5 McNair Medical Institute, Houston, TX 77030, USA; 6 Jan and Dan Duncan Neurological Research Institute, Texas Children’s Hospital, 1250 Moursund Street, Houston, TX 77030, USA

## Abstract

Efficient gene editing is essential to fully utilize human pluripotent stem cells (hPSCs) in regenerative medicine. Custom endonuclease-based gene targeting involves two mechanisms of DNA repair: homology directed repair (HDR) and non-homologous end joining (NHEJ). HDR is the preferred mechanism for common applications such knock-in, knock-out or precise mutagenesis, but remains inefficient in hPSCs. Here, we demonstrate that synchronizing synchronizing hPSCs in G2/M with ABT phase increases on-target gene editing, defined as correct targeting cassette integration, 3 to 6 fold. We observed improved efficiency using ZFNs, TALENs, two CRISPR/Cas9, and CRISPR/Cas9 nickase to target five genes in three hPSC lines: three human embryonic stem cell lines, neural progenitors and diabetic iPSCs. neural progenitors and diabetic iPSCs. Reversible synchronization has no effect on pluripotency or differentiation. The increase in on-target gene editing is locus-independent and specific to the cell cycle phase as G2/M phase enriched cells show a 6-fold increase in targeting efficiency compared to cells in G1 phase. Concurrently inhibiting NHEJ with SCR7 does not increase HDR or improve gene targeting efficiency further, indicating that HR is the major DNA repair mechanism after G2/M phase arrest. The approach outlined here makes gene editing in hPSCs a more viable tool for disease modeling, regenerative medicine and cell-based therapies.

Genetic engineering allows for precise manipulation of the genome, facilitating developmental and disease modeling in accessible experimental systems, which are particularly important in regenerative medicine. Human pluripotent stem cells (hPSCs), including induced pluripotent stem cells (iPSCs) and human embryonic stem cells (hESCs), can give rise to any cell type in the body, including cells affected by disease^1^,^2^. In order to fully utilize the potential of PSC technology, efficient strategies for gene editing in these cells are essential. Classical gene editing approaches based on homologous recombination (HR) have been fruitfully used in mouse embryonic stem cells for decades^3^,^4^,^5^; while successful in principal, these same approaches are extremely inefficient in hPSCs^6^,^7^,^8^.

Recent advances in genetic technology have provided increasingly simpler and more efficient ways to modify the genome based on the generation of double stranded DNA breaks (DSBs) through damage-inducing endonucleases directed by engineered guides to loci of interest. Zinc finger nucleases (ZFNs)^9^,^10^, transcriptional activator-like effector nucleases (TALENs)^11^, and clustered regularly interspaced palindromic repeats (CRISPR)^12^,^13^,^14^ technologies employ modular guides designed by the user to induce DNA damage and increase gene targeting efficiency. With ZFNs, TALENs, and CRISPR, DNA damage can be repaired through non-homologous end joining (NHEJ), leaving an insertion or deletion (indel), or homologous recombination (HR) for homology directed repair (HDR), in which a sister chromatid or template aids in repairing the broken DNA. Both mechanisms of DSB repair — NHEJ and HR — are active in nearly all cell types and species. HR is enriched endogenously during the G2/M phase of the cell cycle^15^. NHEJ is the primary repair mechanism in the G1 phase before DNA synthesis occurs, although it has been detected throughout the cell cycle^15^. When genomic insults such as DSBs occur in hPSCs, damaged cells preferentially undergo apoptosis to limit the replication of compromised DNA and maintain the integrity of the population, leading to a shift away from DNA repair by HR in damaged hPSCs^16^. The result is a decrease in incorporation of homologous template DNA, with effective gene targeting rates oscillating between 0.5–8%^17^ in hPSCs. HDR allows for precise genome modification and is necessary for many common applications such as knock-in of fluorescent reporters, precise mutations, or selection cassettes that are delivered as exogenous DNA fragments, making HDR crucial and thus gene editing challenging. Therefore, tools directing cells to preferentially undertake one route of DNA repair (HR) over the other (NHEJ) could facilitate the desired targeting events.

Improving the rate of HDR will substantially increase the efficiency of genetic engineering. Recent studies have shown that small molecules like SCR7, BrefeldinA, or L755507 can inhibit NHEJ or manipulate the cell cycle; however, these tools have limitations. For instance, they were tested in carcinoma cell lines or mouse embryos, showed toxicity, have not been thoroughly investigated for various endonuclease or gene targeting strategies, have not been tested bi-directionally to alter the cell cycle, or have not been compared to technologies to influence other phases of the cell cycle^18^,^19^,^20^,^21^,^22^. In addition, these studies have only shown the effect of small molecules on targeting efficiencies without delineating the underlying biological mechanism.

Our goal is to find efficient strategies to shift human cells, in particular hPSCs, towards HDR during gene editing using various customizable endonucleases and to improve gene modification efficiency in a locus-independent manner. Here, we systematically determined conditions to increase the effectiveness of precise, template-based repair in genome editing by CRISPR, CRISPR nickase, ZFNs, and TALENs by synchronizing five different hPSC and five different hPSC lines and hPSC-derived cells in the G2/M phase, during which the endogenous repair mechanisms for HR are abundant. We show that the G2/M phase of cell cycle in itself is sufficient to improve targeting efficiencies, with sorted populations of FUCCI-H9 cells showing a robust increase in template repair in G2/M rich populations and a drastic decrease in G1 rich populations. To synchronize cells with small molecules for increased HDR, we sought compounds with low toxicity that arrest cells in the G2/M phase, and have a reversible effect on the cell cycle. In addition, since the most widely targeted cell types are hPSCs, synchronized cells must also maintain their pluripotency and ability to differentiate efficiently into all three germ layers. We identified two compounds that met these requirements: ABT-751 (ABT) and Nocodazole. Both compounds were initially identified as anti-cancer agents and their biological effects on cells are well understood; ABT and Nocodazole inhibit microtubule polymerization, arresting cells in the G2/M phase of the cell cycle^23^,^24^. We determined the influence of these compounds on repair mechanisms and the effects on both indel formation and HDR repair. Further testing was done to asses the combinatorial effect of enhancing HDR with ABT or Nocodazole while concurrently inhibiting NHEJ using SCR7^19^,^21^. Using these compounds, we synchronized in the G2/M phase human embryonic stem cell lines H1-WA01 (H1)^25^, HUES8^26^, and Fucci H9-WA09 (Fucci-H9) and two human diabetic iPSCs (DiPSCs)^27^, and hPSC-derived neural progenitor cells (NPCs). Synchronized cells consistently have a higher amount of HR and the efficiency of targeting is increased 3–5 fold compared to unsynchronized cells. After synchronization, cells remain pluripotent, do not prematurely express markers of differentiated germ layers, and successfully differentiate into multiple lineages.

Through increased HR activity, reversible synchronization of cells in the G2/M phase improves the efficiency of donor integration during genome editing. The ability to increase the efficiency of genome editing was confirmed in multiple cell types using several gene-editing technologies and targeting five different genetic loci, showing the broad consequences of this new and useful tool for regenerative medicine and translational research.

## Results

### Nocodazole and ABT synchronize human pluripotent stem cells in the G2/M cell cycle phase

**Figure 1:**
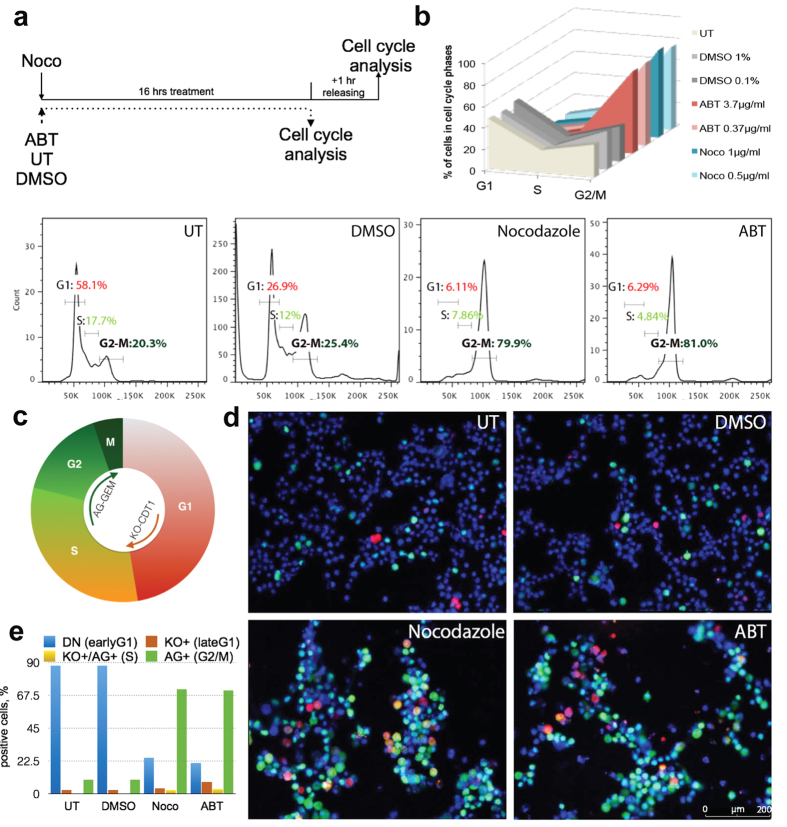
Efficient G2/M cell cycle phase synchronization of hPSCs. (**a**) Treatment timeline and scheme of cell cycle analysis of untreated (UT), vehicle control-DMSO (DMSO), Nocodazole (Noco) and ABT-751 (ABT) treated H1 hESCs. (**b**) Evaluations of different concentrations of DMSO, Noco and ABT. Percentage of cells in G1, G2/M and S phase were determined by flow cytometry by PI staining (upper right panel). Examples of DNA content captured by flow cytometry of untreated (UT), vehicle control-DMSO (0.1%), Nocodazole (1 μg/ml) and ABT-751 (0.36 μg/ml) treated H1 cell. (**c**) Schematic diagram of Fucci system. CDT1 are fused to Azami Green-1 (AG) and GEMININ are fused to Kusabira orange-2 (KO). Double negative (DN) fraction represents early G1 cells, the KO (red) fraction represents late G1, the AG (light green) represents S phase, and the AG high (green) represents G2/M. (**d**) Fucci H9 cells were stained with DNA dye, Hoechst 33342 and analyzed for cell cycle phases by fluorescent microscopy in normal culture conditions and after small molecule-mediated synchronization. (**e**) Quantification of G2/M phase by AG (green), G1 phase by KO (red), S phase by double positive (orange) and early G1 by double negative (blue) cells.

As HR contributes to the maintenance of genomic stability in the G2/M cell cycle phase, we hypothesized that the timely arrest of hPSCs in G2/M phase immediately prior gene targeting would result in increased HR efficiency and donor integration. We tested two compounds to arrest cells in the G2/M phase, Nocodazole and ABT, in three different hPSC lines, H1, HUES8 and Fucci-H9 cells, two human diabetic iPSCs (DiPSCs), and hPSC-derived neural progenitor cells (NPCs). ABT and Nocodazole inhibit microtubule polymerization to arrest the cell cycle, and since their mechanism is well studied the risk of unknown targets in the cell is reduced^23^,^24^. For H1 and HUES8 cell lines, we tested various synchronization periods and releasing combinations to find the optimal conditions to enrich G2/M phase compared to untreated cells and vehicle control (DMSO) (Fig. 1 and Supplementary Tables 1 and 2). Furthermore, we examined dosage effects and cytotoxicity of ABT and Nocodazole on hPSCs. Detailed optimization of both the concentration of compound and the length of induction, as well as the proper adjustment of cell seeding densities, led to the efficient enrichment in G2/M phase for all tested cell lines. A 16-hour treatment with 1 μg/ml Nocodazole and one hour of releasing in Nocodazole-free medium (Fig. 1a) shows robust G2/M cell phase enrichment with 80% of surviving cells in G2/M phase (Fig. 1b and Supplementary Table 1). At the same time, the majority (58.1%) of untreated cells were in G1 phase (Fig. 1b and Supplementary Table 1). 16 hours of ABT treatment had similar results, with 80% of surviving cells synchronized in the G2/M phase (Fig. 1b and Supplementary Table 2).

The fluorescent ubiquitination cell cycle indicator, Fucci-H9 hPSCs, encodes two colors, red fluorescence in late G1 phase and Azima green (AG) in G2/M phase, allowing us to follow cell division and further characterize and quantify cell cycle progression in the presence or absence of Nocodazole or ABT (Fig. 1c,d)^28^,^29^,^30^. Fucci-H9 cells were synchronized by the optimized protocol and fluorescence reflecting G2/M and other cell cycle phases was detected using fluorescent microscopy (Fig. 1d) and flow cytometry (Fig. 1e and Supplementary Figs 1 and 2). After synchronization with Nocodazole or ABT, 88.4% and 88.6% of total live cells respectively, expressed AG as detected consistently by flow cytometry and fluorescent microscopy, indicating a 2.5-fold increase in G2/M phase compared to untreated (38.23%) or DMSO-treated cells (34.4%) (Fig. 1d,e, and Supplementary Figs 1 and 2). Lastly, Fucci-H9 cells were stained using Hoechst DNA dye to further determine each cell cycle phase and analyzed by flow cyometry (Supplementary Fig. 1). The dye confirmed the enrichment of cells in G2/M phase induced by Nocodazole or ABT.

Finding factor(s) that arrested hPSCs in G2/M in a reversible manner without any loss in self-renewal potential was crucial. Therefore, we investigated cell survival after synchronization to determine immediate cytotoxicity induced by Nocodazole or ABT and then followed cell proliferation after releasing for 24 and 48 hours (Supplementary Fig. 3). Approximately 40% of hPSCs synchronized with either Nocodazole or ABT, survive and proliferate at normal rates between 24 and 48 hours after compound removal, showing the synchronization is reversible and does not have a long-term deteriorating effect on hPSCs.

### Cell cycle synchronization increases global gene repair, predominantly HDR

**Figure 2:**
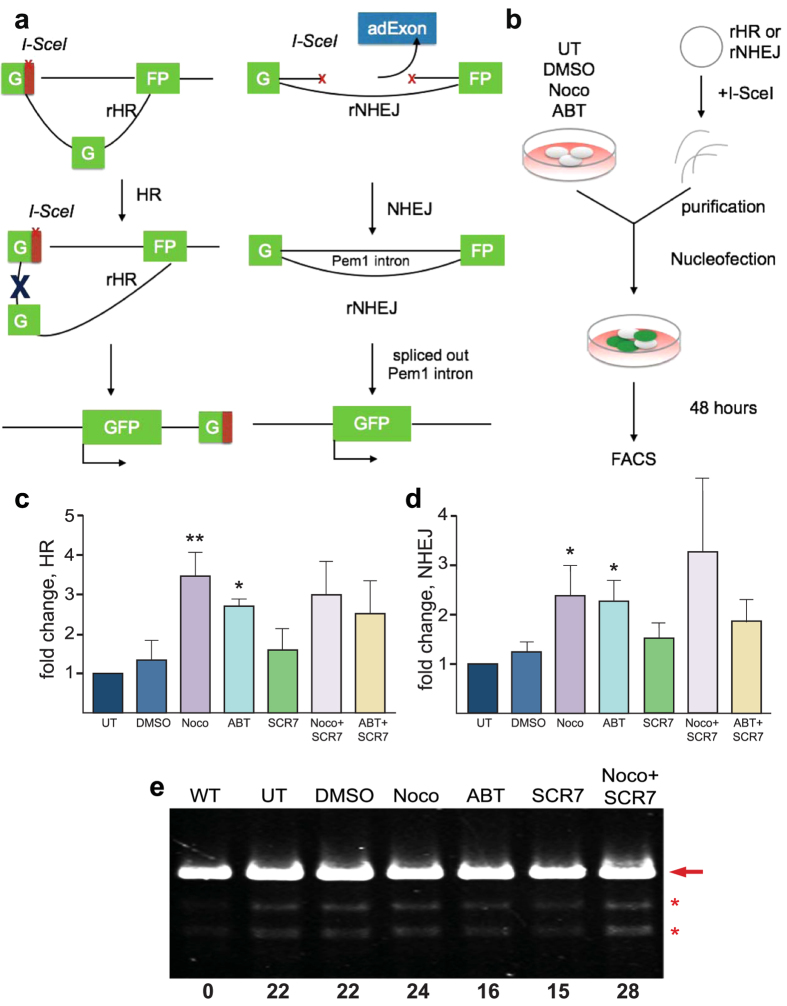
HR and NHEJ activity assessment after G2/M synchronization in hPSCs. (**a)** Schematic diagram of the mechanism of rHR (left) and rNHEJ (right) reporter. **(b)** Experimental design of HR and NHEJ analysis after synchronization or treatment of Noco, ABT, SCR7, Noco with SCR7, control DMSO and untreated H1 cells. H1 cells were nucleofected with digested rHR and rNHEJ and the percentage of GFP positive cells was captured by FACS for determining **(c)** HR and **(d)** NHEJ activity in synchronized cells normalized to untreated (n = 3 biological replicates, **P < *0.01* for Noco and *P < *0.05* or ABT). **(e)** Indel formation after CRISPR/Cas9 targeting at the *OCT4* locus in synchronized and control HEK293t cells using T7 endonuclease assay. Error bars represent SEM.

To analyze the extent to which cell synchronization alters the repair system in the cell, we measured the efficiency of HR and NHEJ in a quantitative manner. We used a fluorescent HR reporter (rHR) and NHEJ reporter (rNHEJ)^31^, in which a functional *GFP* gene is reconstituted following an HR or NHEJ event (Fig. 2a). H1 cells were treated with ABT or Nocodazole or were transfected with the HR or the NHEJ reporter linearized by digestion with *I-SceI* enzyme (Fig. 2b). After transfection, cells were incubated for 48 hours to allow for the expression of GFP and were analyzed by flow cytometry. GFP detection showed a 2–4 fold increase in HR and NHEJ after G2/M phase synchronization compared to untreated cells (0.43%) (Fig. 2c). Synchronization with Nocodazole resulted in a 3.5-fold increase in HR (1.5%), and ABT treatment had a 3.1-fold increase (1.35%) (Fig. 2c). NHEJ activity increases by about 2-fold in Nocodazole (2.75%) and ABT (2.6%) synchronized cells, compared to non-synchronized cells (1.10%) (Fig. 2d). We also used SCR7, an inhibitor of DNA ligase IV necessary for canonical NHEJ pathway, to test whether transiently blocking NHEJ enhances the frequency of HR (Fig. 2c–e)^19^,^21^. SCR7 treatment alone or in combination with Nocodazole or ABT did not show further increase of HR or change in NHEJ activity (Fig. 2c,d).

Targeting HEK293t cells with CRISPR/Cas9 to create a DSBs at the *OCT4* locus and evaluating NHEJ at the functional level reveals no change in indel formation between Nocodazole synchronized and non-synchronized cells, but 30% decrease in indel for ABT synchronized cells (Fig. 2e and Supplementary Fig. 4). Similarly, a 30% decrease in indel was detected for SCR7 treated cells (Fig. 2e and Supplementary Fig. 4).

**Figure 3:**
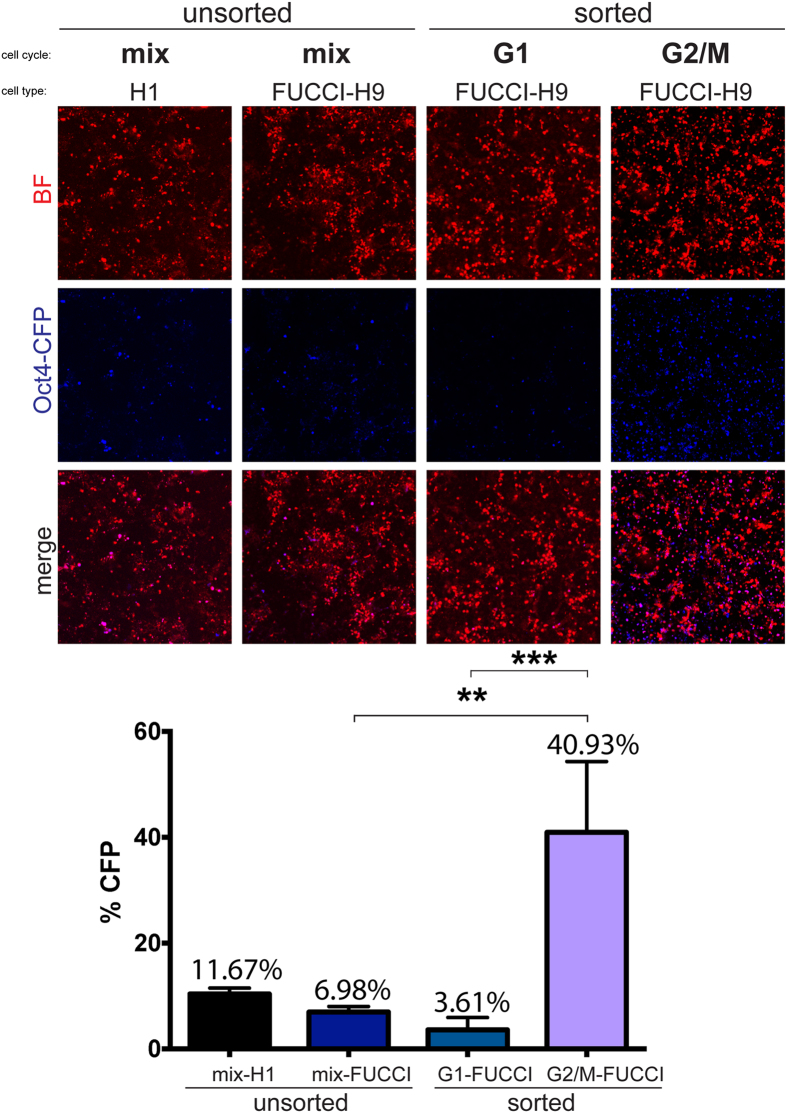
Targeting efficiency shifts depending on the cell cycle with an increase in G2/M populations and a decrease in G1 populations. Five days after FACS sorting and CRISPR *S. pyogenes* targeting to insert CFP into the *OCT4* locus, unsorted H1 hPSCs, unsorted FUCCI-H9 cells, sorted FUCCI-H9 in the G1 phase, and sorted FUCCI-H9 in the G2/M phase were imaged by confocal microscopy for CFP expression. Brightfield (BF); Cyan Fluorescent Protein (CFP) (n = 3 biological replicates, **P < 0.01; ***P < *0.001*).

**Figure 4:**
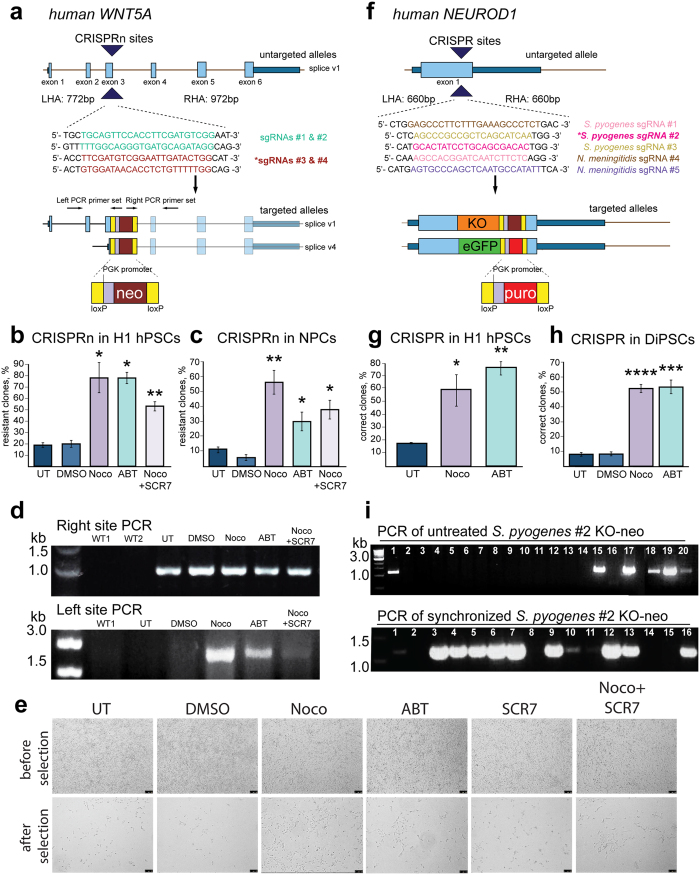
G2/M synchronization increases gene targeting efficiency of *WNT5A* by CRISPR/Cas9 nickase and *NEUROD1* by two different CRISPR/Cas9 species. (**a**) Human *WNT5A* gene structure and targeting scheme. *WNT5A* is targeted for knockout with a floxed neomycin cassette. *indicates sgRNA used for subsequent panels. (**b**) The efficiency of targeting in H1 cells determined by counting the percent of colonies surviving after antibiotic selection; *n* = 2 independent experiments. *P < *0.05*; **P < 0.01; ****P < *0.0001* (**c**) The efficiency of targeting in H1-derived NPCs determined by counting the percent of cells surviving after antibiotic selection; *n* = *2* biological replicates. *P < *0.05*; **P < *0.01* (**d**) PCR confirmation of targeted alleles. (**e**) Brightfield images of NPCs after targeting and before selection in top row and after targeting and after selection for neomycin resistance in the bottom row. (**f**) Human gene structure of *NEUROD1* and targeting scheme. *indicates sgRNA used for subsequent panels. (**g**) The efficiency of targeting in H1 cells by counting percent of correct colonies compared to resistant colonies determined by 3′, 5′ end and internal PCRs; *n* = 3 independent experiments. *P < *0.05*; **P < *0.01.* (**h**) The efficiency of targeting in DiPSCs by counting percent of correct colonies compared to resistant colonies determined by 3′, 5′ end and internal PCRs, *n* = *4* independent experiments. ***P < 0.001; ****P < *0.0001* (**i**) PCR confirmation of the identity of targeted alleles in untreated clones in the top panel or Nocodazole-synchronized cells in bottom panel. Gene structure is annotated including exons (light blue), introns (brown lines), untranslated regions (dark blue), and targeting sites (large navy arrows). Below the untargeted allele(s) the target sequence is shown with left homology arm (LHA) and right homology arm (RHA) sizes. Targeted alleles show the donor cassette integrated into the correct locus. Kusabira orange (KO) and eGFP cassettes have 3′ antibiotic resistance genes, KO with neomycin and eGFP with puromycin. Error bars represent SEM.

**Figure 5:**
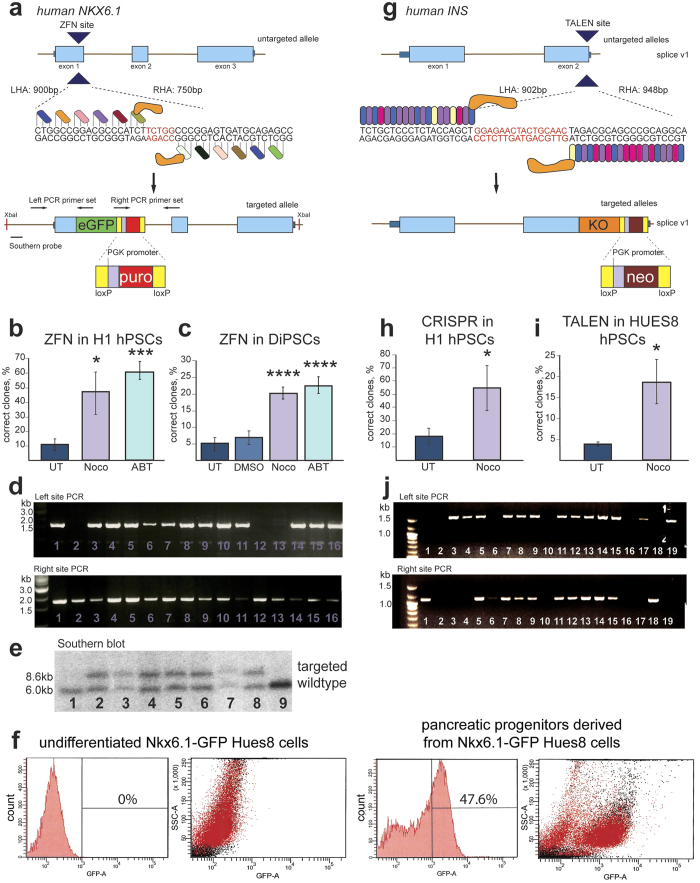
G2/M synchronization increases targeting efficiency of *NKX6.1* with ZFNs and *INS* with TALENs and CRISPR/Cas9. (**a)** Human *NKX6.1* gene structure and targeting scheme. ZFNs cleave using FokI nuclease at the 3′ end of the first exon to create DSB for insertion of a fluorescent reporter, eGFP. **(b)** The efficiency of targeting in H1 cells determined by counting the percentage of correctly targeted colonies compared to resistant colonies after PCR confirmation; *n* = 2 independent experiments. *P < *0.05*; ***P < *0.001* **(c)** The efficiency of targeting in DiPSCs determined by counting the percentage of correctly targeted colonies compared to resistant colonies after PCR confirmation; *n* = 2 independent experiments. ****P < *0.0001.* **(d)** PCR confirmation of the identity of targeted individual numbered clones. **(e)** Southern blot confirmation of targeted alleles after XbaI digestion and 5′ end probe external to the integrating cassette. Correctly targeted clones are 8.6 kb while the wildtype clones are 6.0 kb. **(f)** GFP is not expressed in undifferentiated NKX6.1-GFP HUES8 cells and is unregulated specifically in pancreatic progenitors confirming the faithfulness of the reporter. **(g)** Human gene structure of *INS* and targeting scheme. *INS* was targeted using TALENs with FokI nuclease to create a DSB in the 3′ end of the gene for insertion of a fluorescent reporter, KO. **(h)** The efficiency of targeting in H1 cells determined by counting the percentage of correctly targeted colonies compared to resistant colonies after PCR confirmation; *n* = 3 independent experiments. *P < *0.05.* **(i)** The efficiency of targeting in HUES8 cells determined by counting the percentage of correctly targeted colonies compared to resistant colonies after PCR confirmation; *n* = 3 independent experiments. *P < *0.05.* **(j)** PCR confirmation of the identity of targeted individual numbered clones. Gene structure is annotated including exons (light blue), introns (brown lines), untranslated regions (dark blue), and targeting sites (large navy arrows). Below the untargeted allele(s) the target sequence is shown with left homology arm (LHA) and right homology arm (RHA) sizes. Targeted alleles show the donor cassette integrated into the correct locus. Kusabira orange (KO) and eGFP cassettes have 3′ antibiotic resistance genes, KO with neomycin and eGFP with puromycin. Error bars represent SEM.

To further examine the effects of synchronization and the stress of nucleofection on DNA repair mechanisms, we detected by qPCR acute HR- and NHEJ-related gene expression, including *BRCA1, BRCA2, RAD51* for HR, and *LIG4, XRCC4, XRCC5* for NHEJ, at 3, 6, 12 and 24 hours after transfection. We found no significant differences between Nocodazole-, ABT-treated and untreated cells, indicating that effects on transcriptional machinery did not increase the levels of HR and NHEJ (Supplementary Fig. 5). While the reporter assay shows a 3.5-fold increase in HR (Fig. 2b) and a 2-fold increase in NHEJ (Fig. 2c) with Nocodazole or ABT, functional assays show that synchronizing hPSCs with Nocodazole or ABT increases HR (Figs 3, 4, 5) with little effect on indel formation, or NHEJ at the functional level (Fig. 2d).

### Synchronization of multiple human cell lines in G2/M phase increases on-target gene editing efficiency using CRISPR, CRISPRn, ZFNs, and TALENs for five different genetic loci

To test the efficiency of on-target gene editing after cell synchronization in the G2/M phase, we used customized nuclease gene targeting systems such as, ZFNs, TALENs, CRISPR and CRISPRn to target five genes in five different human PSC lines (Table 1). To first determine if cell cycle phase increases the efficiency of correct targeting, we FACS sorted FUCCI-H9 cells based on their cell cycle indicators to obtain G1 and G2/M enriched populations. These populations were immediately targeted after sorting with CRISPR/Cas9 *S. pyogenes* to incorporate Cyan Fluorescent Protein (CFP) at the 3′ end of the *OCT4* loci, a gene that is known to be accessible for targeting in hPSCs^6^,^11^. Five days post targeting, G2/M populations showed robust CFP expression with 40.93% CFP+ cells out of total cells, compared to 6.98% for unsorted, nonsynchronized, FUCCI-H9 or 11.67% for H1 hPSCs (Fig. 3). G1 populations showed decrease CFP expression (3.61%) compared to unsorted FUCCI-H9. The significant increase in CFP+ cells targeted in G2/M phase and decrease in G1 phase, respectively, indicates that cell cycle arrest influences gene editing efficiency and synchronization of cells with Nocodazole or ABT may be a useful tool to increase the efficiency of these technologies.

We next used CRISPR *S. pyogenes* D10A nickase to target the *WNT5A* locus, a gene high in heterochromatin content compared to Oct4, to create a knockout by inserting a neomycin resistance gene into the first constitutive exon of *WNT5A* (Fig. 4a). Nocodazole or ABT synchronization again significantly increased the number of clones with integrated donor cassette as measured by % of individual clones before and after antibiotic selection (78.43% and 78.41%, respectively), compared to untreated, non-synchronized cells (18.65%) or cells treated with the vehicle/DMSO (20.14%) (Fig. 4b). While combining Nocodazole with SCR7 had a significant increase in resistant clones compared to control cells (53.0%), this combination did not further improve targeting compared to Nocodazole treatment alone. We tested this approach on H1-derived NPCs, which are known to have a higher rate of NHEJ than hPSCs^32^, and found that they were also more susceptible to targeting after synchronization in the G2/M phase of the cell cycle. Nocodazole (56.3%) and ABT (29.67%) treatment increased the number of cells resistant to antibiotic treatment compared to untreated cells (11.03%) and DMSO-treated cells (5.57%) (Fig. 4c,e). Again, combining Nocodazole with SCR7 showed increased gene-targeting efficiency (37.86%) when compared to control, but at a lower level than with Nocodazole alone. Donor integration was confirmed by PCR of the right site of the cassette and the left site of the cassette, showing integration of the neomycin cassette in the *WNT5A* locus in targeted H1 hPSCs (Fig. 4d). Targeting of *WNT5A* in hPSCs and NPCs shows that synchronization has a positive effect on the efficiency of genome modification in various human cell types.

To investigate whether synchronization improves the cassette insertion efficiency at any given gene, we chose to target human *NEUROD1* in non-synchronized and Nocodazole-, and ABT-synchronized H1 hPSCs using CRISPR *S. pyogenes* and *N. meningitidis* to create a knock-in of fluorescent genes (Kusabira orange, KO, or eGFP) with an antibiotic resistance gene (Fig. 4f). We tested both the commonly applied CRISPR *S. pyogenes* as well as CRISPR *N. meningitidis*, as CRISPR *N. meningitidis* has been shown to have a higher targeting efficiency in hPSCs than CRISPR *S. pyogenes* and TALENs^33^. Cells synchronized with Nocodazole (59.77%) or ABT (79.91%) showed a 3–5 fold increase in the number of clones with targeting cassette integrated at the 3′ of *NEUROD1* exon1 with compared to untreated cells (17.46%), with CRISPR *S. pyogenes* targeting quantified by % of single clones positive for three independent PCRs confirming the correct targeting out of total analyzed clones (n > 200) (Fig. 4g). These data suggest that improvements in on-target insertion efficiency after elongation of the G2/M phase are not limited to a particular gene. We then targeted DiPSCs, as iPSCs are increasingly used for regenerative medicine studies, using CRISPR *N. meningitides* and again observed a significant increase in on-target donor integration when cells were synchronized with Nocodazole (52.49%) or ABT (53.50%) compared to untreated (7.66%) and DMSO (7.86%) controls (Fig. 4h). Donor integration in each experiment was confirmed by PCRs for individual clones in untreated and synchronized cultures, verifying the integration of the donor cassette in the correct locus and showing a higher efficiency of correctly targeted clones in synchronized cells (Fig. 4i).

Although CRISPR is currently the most popular targeting strategy, we were interested in determining whether the effect of arresting cells in G2/M phase was unique to this technology or if other DSB-based gene editing tools would also benefit from synchronization. We targeted H1 and Hues8 hPSCs and DiPSCs for knock-in of eGFP fluorescent reporter with a floxed puromycin resistance gene in the *NKX6.1* locus with ZFNs (Fig. 5a). Synchronization of H1 and Hues8 hPSCs with Nocodazole (47.19%) or ABT (60.46%) showed 4 to 6-fold increases in efficiency of donor integration at the 3′ end of *NKX6.1* exon 1 versus untreated cells (9.55%) (Fig. 5b). Synchronization of DiPSCs with Nocodazole and ABT also increased desired gene editing efficiency with ZFNs, 20.4% and 22.95% respectively, compared to untreated (5.23%) and DMSO-vehicle treated (6.95%) cells (Fig. 5c), as quantified by % of correctly targeted clones out total analyzed clones. PCR was used to confirm the correct insertion of the donor cassette into the *NKX6.1* exon 1 by amplifying the left insertion site and right insertion site, and selected clones were further verified by Southern blot using external 5′ end probe (Fig. 5d,e). Since *NKX6.1* is not expressed in the hPSC stage, we additionally confirmed the functionality of the GFP reporter by *in vitro* differentiation of NKX6.1-eGFP Hues8 clones into pancreatic progenitors and analyzed eGFP fluorescence by flow cytometry. The GFP was not expressed in undifferentiated cells but specifically up regulated at pancreatic progenitors, when 47.6% of total cells expressed eGFP (Fig. 5f). Finally, we targeted H1 and Hues8 cells using CRISPR *S. pyogenes* and TALENs to tag human *INSULIN* with a fluorescent KO reporter and a neomycin resistance gene (Fig. 5g). Targeting untreated, non-synchronized H1 cells with CRISPR resulted in 17.77% clones positive for two external PCR confirming the correct integration of KO and selection gene, out of total analyzed clones (n = 300), compared to 54.64% for Nocodazole-treated cells (Fig. 5h). Hues8 hPSCs targeted with TALENs to the same locus showed a 5.33-fold increase in *INSULIN* targeting efficiency after synchronization with Nocodazole (Fig. 5i) compared to DMSO treated controls, showing that the increased efficiency of targeting after synchronization is independent of targeting method since both CRISPR and TALEN targeting at the same human *INSULIN* locus was markedly improved. Correct integration was confirmed by 5′ and 3′ external PCRs as well as cassette specific PCR and Southern Blot (Fig. 5j and data not shown). By targeting different human loci with different donor cassettes in various hPSC lines and hPSC-derived differentiated cells using ZFNs, TALENs, CRISPR *S. pyogenes*, CRISPR *N. meningitides*, and CRISPR *S. pyogenes* D10A nickase in synchronized and unsynchronized cells, we have shown that gene editing is globally improved by synchronization of the cell cycle at the G2/M phase. This improvement is an advance towards high efficiency gene editing in hPSCs and other human cell types.

### hPSCs retain pluripotency and are capable of differentiating into different germ layers upon G2/M synchronization

**Figure 6:**
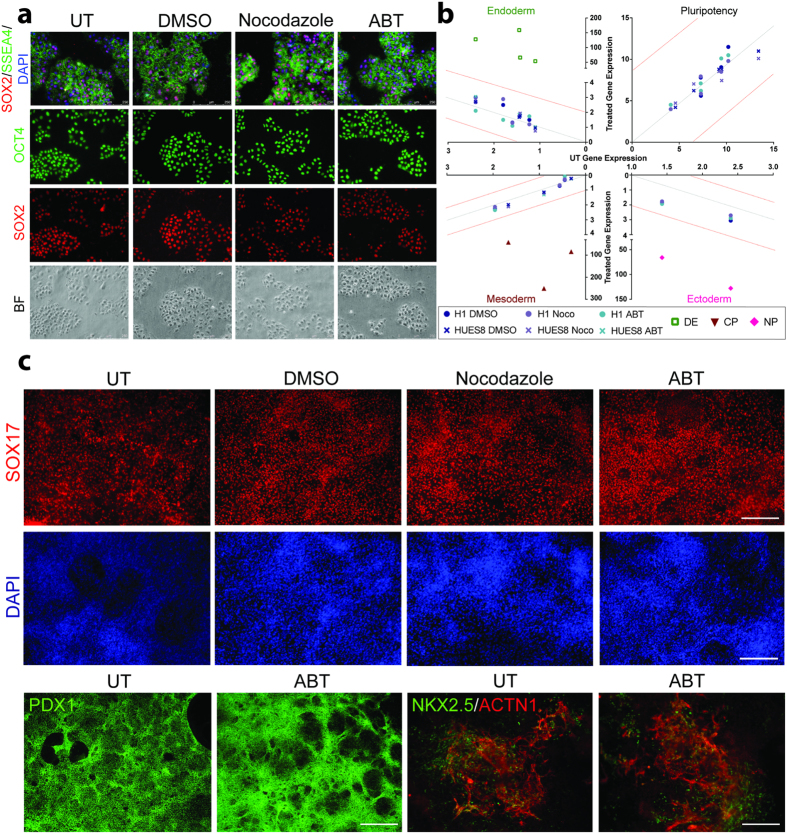
Pluripotency is maintained in hPSCs after synchronization. (**a)** H1 hPSCs were synchronized with Nocodazole, ABT, the vehicle DMSO, or left untreated before staining for markers of pluripotency, including SSEA4, SOX2, and OCT4. The bottom panel is bright-field imaging showing that all cells express OCT4 and SOX2. **(b)** Gene expression analysis by qPCR of 5 pluripotency markers, 4 markers of endoderm, 3 markers of mesoderm, and 2 markers of ectoderm in Nocodazole- and ABT-synchronized and DMSO-treated H1 and HUES8 hPSCs compared to untreated cells. Correlation of *R* = *1.*0 shown by a dashed gray line. Dashed red lines annotate the 95% confidence interval from untreated gene expression, with all markers within these two lines not statistically different from untreated cells. H1 and HUES8 cells were differentiated into definitive endoderm (DE), cardiac progenitors (CP), and neural progenitors (NP) to show the fidelity of candidate markers of germ layers. Target genes and expression is shown in Supplementary Table 11 (H1 hPSCs marked by circles, HUES8 by X, DMSO by dark blue, Nocodazole by purple, ABT by light blue, DE by green, CP by red, and NP by pink). **(c)** Synchronized and untreated H1 hPSCs differentiated into pancreatic progenitors (PDX1; upper left), cardiac progenitors (NKX2.5/ACTN1; upper right), and definitive endoderm (SOX17, lower).

Cell cycle manipulation and G0/G1 phase elongation have been shown to stimulate the differentiation of hPSCs^30^,^34^,^35^. To explore whether arrest in the G2/M phase stimulates hPSC differentiation, immunofluorescence staining was performed on synchronized H1 hPSCs with pluripotency markers, such as SSEA4^36^, OCT4^37^, and SOX2^38^ (Fig. 6a). All cells stimulated with either Nocodazole or ABT exhibited expression of known markers of pluripotency and tri-lineage differentiation potential. Gene expression analysis of pluripotent genes *OCT4, NANOG*^38^*, REX1*^39^*, GDF3*^40^, and *LIN28*^41^ in Nocodazole- and ABT-synchronized hPSCs showed strong correlation to untreated cells (Fig. 6b and Supplementary Table 11; H1 DMSO *R*^*2*^ = 0.83, Nocodazole *R*^*2*^ = 0.89, ABT *R*^*2*^ = 0.93; Hues8 DMSO *R*^*2*^ = 0.97, Nocodazole *R*^*2*^ = 0.90, ABT *R*^*2*^ = 0.97). Additionally, there was no difference between synchronized and unsynchronized hPSCs in expression of endoderm markers, such as *FOXA2*^42^*, GATA4*^43^*, CXCR4*^44^, and *SOX17*^45^ (Hues8 DMSO *R*^*2*^ = 0.94, Nocodazole *R*^*2*^ = 0.88, ABT *R*^*2*^ = 0.96; definitive endoderm *R*^*2*^ = 0.25); mesoderm markers, such as *BRA(T)*^46^*, TPNT*^36^, and *NKX2.5*^47^ (H1 DMSO *R*^*2*^ = 0.98, Nocodazole *R*^*2*^ = 0.98, ABT *R*^*2*^ = 0.97; Hues8 DMSO *R*^*2*^ = 0.99, Nocodazole *R*^*2*^ = 0.99, ABT *R*^*2*^ = 0.97; cardiac progenitors *R*^*2*^ = 0.07), and ectoderm markers, such as *PAX6*^48^ and *SOX1*^49^ (Fig. 6b and Supplementary Table 11). Quantitative PCR results confirmed that *in vitro* differentiated iPSCs expressed markers of definitive endoderm, cardiac progenitors, and neural progenitors (Fig. 6b). Genome editing of hPSCs could be applied to model human diseases or to make patient-specific lines to correct patient mutations and must not affect the differentiation potential to generate necessary cell types of any lineage. Therefore, we determined the differentiation potential of synchronized hPSCs into endoderm, pancreatic progenitors, and cardiac progenitors. After synchronization, H1 hPSCs differentiate into endoderm (SOX17), pancreatic progenitors (PDX1), and cardiac progenitors (NKX2.5 and ACTN1) (Fig. 6c). Collectively, these results show that transient G2/M arrest does not affect the pluripotency of hPSCs or their morphology, and they maintain their potential to differentiate into multiple germ layers, making synchronization a useful tool for improving the efficiency in human genome editing.

## Discussion

We report a simple and robust gene targeting system to increase donor integration by enriching HR events. For the first time, we demonstrate ABT and Nocodazole as separate tools to efficiently synchronize hPSCs in the G2/M cell cycle phase with minimal cytotoxicity. This synchronization leads to an increase in HDR events and subsequently significantly improves on-target genome engineering efficiency. Pluripotency is not affected after G2/M phase synchronization and cells remain capable of differentiation into multiple lineages, making ABT and Nocodazole great tools for gene editing in hPSCs. Using ZFNs, TALENs, two different species of CRISPR/Cas9, and CRISPR nickase, we successfully targeted five different human cell lines for five different genes. We observed a locus-independent increase in efficiency of on-target gene targeting after synchronization in four different hPSCs as well as differentiated multipotent human cells (such as NPCs). Additionally, synchronization equally improved efficiency of all four targeting tools used to induce DSBs, demonstrating that increased HDR after G2/M phase arrest is a robust and universal property of hPSCs.

During the G2/M phase, cells preferentially repair DNA by HR when sister chromatids are available to act as homologous templates^15^. The principal repair mechanisms of hPSCs at different phases of the cell cycle are not well-established^32^,^50^. To understand the mechanism of repair in hPSCs during the G2/M phase, we synchronized cells to test the rate of HR and NHEJ. hPSCs arrested in the G2/M phase had elevated levels of HR compared to untreated hPSCs. In particular, ABT synchronization induces high rates of HR and decreased levels of indel formation compared to untreated cells. Therefore, ABT might be the optimal choice to improve the efficiency of gene targeting.

Other small molecules have been tested to enhance gene targeting either in mouse stem cells or embryos as well as in human carcinoma cell lines, but these studies focused only on CRISPR, showed some toxicity or were technically challenging^18^,^19^,^20^. To further improve efficiency, with ABT and Nocodazole we also tested SCR7, which acts to inhibit NHEJ through interference of DNA binding in the canonical NHEJ pathway^19^. When we combined Nocodazole or ABT with SCR7, we observed some decrease in NHEJ rates in hPSC, but found no significant increase in HR or gene targeting efficiency. The negligible effect of SCR7 may indicate that HR is the major DNA repair mechanism in the G2/M phase of hPSCs, with the rate of HR unaffected by further inhibition of NHEJ. In differentiated hESC-derived NPCs, in which NHEJ is 1.3× more predominate than in hPSCs, we again found no additional effect on HDR with SCR7^32^. In hPSCs and NPCs, the canonical NHEJ pathway is the primary mechanism of NHEJ repair, but in circumstances when the canonical pathway is blocked, noncanonical NHEJ pathways independent of DNA ligase IV aid in repair^21^,^32^. While studies have shown inhibiting NHEJ can increase HDR^21^, in hPSCs we found that increasing HDR to prevent NHEJ compensation through noncanonical pathways more effectively increases the efficiency of on-target gene editing by customizable endonucleases.

We found that synchronizing hPSCs in the G2/M cell cycle phase increases the efficiency of gene editing on average 4-fold (Table 1). Enriching the G2/M phase cells to increase gene editing efficiency in hPSCs is not limited to selectable constructs and can also be applied in non-selectable contexts, such as scarless introduction of mutations such as SNPs. The novel tools explored in this report facilitate efficient gene editing of hPSCs, providing the opportunity for human cells to be used for disease modeling, epitope-tagging or fluorescent reporter generation and for patient-derived mutation-corrected tissue for therapeutic application.

## Methods

### Cell culture

hPSC lines, H1, Hues8, and FUCCI-H9, were cultured under feeder-free on hESC-qualified Matrigel (BD Biosciences) in E8 media (Stemcell Technologies) with 30% of irradiated mouse embryonic fibroblasts (iMEFs) conditional media. FUCCI-H9 hPSCs were kindly provided by S. Dalton^29^ and were maintained under G418 sulfate (Sigma, 200 ug/ml) and Puromycin (0.1 ug/ml) selections. Cells were passaged every 3–5 days at 80% confluent with TrypLE Express (Invitrogen). After dissociation, cells were plated in E8 media with 10 uM Y-27632, (StemGent) for 24 hrs. After 24 hrs media, without Y-27632, was replenished daily. iMEF conditional media was prepared by incubating iMEFs with hPSC media without bFGF for 24 hrs for 7 days. Collected media was filtered, flash frozen and stored at −80 ^o^C.

To maintain neural progenitors, cells were cultured in NPM media: DMEM/F12: Neurobasal media at a 1:1 ratio +0.5xB27 +0.5xN2 + 2 mM Glutamax +20 ng/mL bFGF (R&D Systems) +20 ng/mL EGF (R&D Systems) and media was replenished every four days. To split cells, cells were washed with PBS and incubated for 5 min at 37 ^o^C in Accutase (Innovative Cell Technologies). Cells were washed, spun down at 1000 g for 5 min, and replated in NPM media on Geltrex-coated plates.

### Cell cycle synchronization

Concentration and time course of different G2/M synchronization and release times was performed as presented in Supplementary Table 2 and for gene targeting and other experiments the 16 hr incubation and 1 hr release for Nocodazole (Sigma) at 1 ug/ml and 16 hr treatment without release for ABT (Selleckchem) at 0.37 μg/ml was selected. As negative control, solvent for small molecules, DMSO was used at 1:100 or 1:1000 dilution.

### Cell cycle analysis

For DNA content analysis, hPSCs cells were dissociated by TrypLE Express (Invitrogen) and fixed with 70% EtOH. Propidium iodide was used to for H1 and HUES8 cells and Hoechst 33342 (both Sigma) for FUCCI-H9 cells for 30 min at 37 ^o^C to stain DNA. Samples were then fixed with 2% Paraformaldehyde/PBS (PFA), washed and filtered through 40 um cell strainer (BD Biosciences) before flow cytometry. FACS analysis was performed using LSRII (BD Biosciences) and analyzed using FACSDiva software. Gates were set with reference to negative controls.

### FUCCI cell sorting

Cells were sorted on the FACSAria (BD Biosciences) using FACSDiva software.

Gates were set with reference to negative controls. The sorting speed was adjusted to ensure sorting efficiency above 90%. Cells were collected in tubes with Sorting buffer (PBS, 5% FBS and 2 mM EDTA).

### HR and NHEJ reporter assay

The HR and NHEJ reporter constructs were kindly provided by V. Gorbunova (University of Rochester). The plasmids were digested overnight with *I-SceI* at 37 °C and confirmed by gel electrophoresis. Digestion products were then purified by phenol/chloroform extraction. Three μg of digested DNA was nucleofected to 1×10^6^ of H1 cells using CA137 program (Lonza). After incubation for 48 hrs at 37 °C, cells were dissociated and GFP (indicating successful HR or NHEJ repair) was detected and quantified by flow cytometry.

### DNA delivery

hPSCs and NPCs were pre-treated with 10 uM Y-27632, 24 hrs before nucleofection. On the day of nucleofection, hPSCs were disassociated into single cells with TrypLE; Accutase was used for NPCs. 1–2.5 million cells were re-suspended in 100 ul P3 buffer plus Supplement with 10 ug of total DNA and nucleofected using CM113 program using Amaxa 4D-Nuclefector (Lonza). Cells were immediately transferred with 500 ul of appropriate media to 1.5 ml tube and recovered for 10 min at 37 ^o^C before plating on iMEFs at 15,000-cell/cm^2^ density in media with Y-27632.

For indel analysis 293HT cells were treated with DMSO, Nocodazole, ABT, Nocodazole with SCR7, SCR7, or untreated for 16 hrs. Cells were transfected with Lipofectamine 2000 (Life Technologies) with 1.5 ug of pX330 OCT4 sgRNA. Four days after transfection, cells were collected, spun down at 2000 g for 5 minutes, and DNA was extracted.

### Generation of gene targeting and CRISPR-Cas9 Constructs

Single stranded oligonucleotides were phosphorylated and annealed (Supplementary Table 5) before digestion and ligation of pX335 D10A Cas9 plasmid (Addgene #42335) for CRISPRn, pX330 plasmid for CRISPR targeting OCT4, pX459 plasmid for *S. pyogenes* Cas9 targeting of *NEUROD1* (Addgene #48139), or pSimpleII backbone for *N. meningitidis* (Addgene #47868) with paired oligos performed as described^51^ with digestion/ligation with BbsI and T7 ligase by PlasmidSafe exonuclease digestion (EpiCentre).

*WNT5A* was targeted within the first constitutive exon (Supplementary Table 6) using plasmids shown in Supplementary Table 4, with homology arms as shown in Supplementary Table 7. *NEUROD1* was targeted with two different Cas9 species (Supplementary Table 4) with homology arms for the same genomic region (Supplementary Tables 5–7). *NKX6.1* was targeted in the 3′ end using Zinc finger nucleases with FokI endonuclease (Supplementary Tables 4–6). ZFNs were designed to bind to 18 bp flanking a 5 bp spacer sequence using CompoZr^TM^ Custom Zinc Finger Nucleases (Sigma Aldrich) to construct PZFN plasmid DNA as well as *NKX6.1* ZFN mRNA. Activity of *NKX6.1* ZFN plasmid DNA and mRNA was assayed by PCR amplification of the target region with AccuPrimer GC Rich DNA Polymerase (Sigma Aldrich) followed by CelI nucleotide mismatch assay. *INSULIN* was targeted at the 3′ end of the gene with TALENs with FokI (Supplementary Tables 4–7). TALENs were designed using GeneArt^®^ Precision TALs (Life Technologies) and cloned into aTAL entry clones before recombination into the destination vector pcDNA^TM^-DEST40 by Gateway^®^ cloning catalyzed by LR Clonase^®^ II enzyme (Life Technologies). PCR of eCFP using high fidelity Q5 polymerase with primers with a 5′ NheI overhang and 3′ AscI overhang was used to create OCT4-eCFP donor (Supplementary Tables 4–8). All oligonucleotides and homology arms are from IdtDNA. All enzymes unless otherwise noted are from NEB.

### Indel analysis

PCR was performed on DNA using Q5 High Fidelity Polymerase (NEB) to amplify the targeted region (primers in Supplementary Table 8) before isolating by gel extraction. A hybridization reaction with NEBuffer 2, 200 ng PCR product, and water was run at 95 °C for 5 minutes, ramping down to 85 °C at 2 °C/s, ramping down to 25 °C at 0.1°C/s. Hybridized PCR products are incubated at 37 °C with T7 endonuclease I for 15 minutes and then EDTA was used to stop the reaction. Products are run on 6% polyacrylamide gel to distinguish cut and uncut bands.

### Drug selection and clonal expansion

hPSC clones that have stably integrated the targeting vectors were identified through the selection with 50 ug/ml G418 or 0.1 ug/ml puromycin. Selection was started at day 2 post-nucleofection and at day 4 the G418 concentration was increased to 100 ug/ml and puromycin to 0.5 ug/ml. After 7 days of selection, drug-resistant colonies were individually picked into 96-well plates with iMEFs and expanded for cell banking and DNA extraction. Cells for genomic DNA extraction were cultured on 0.1% gelatin coated plates until >90% confluent. A similar procedure was used to identify drug-resistant NPCs.

### Identification of targeted clones

Genomic DNA was isolated using DNaesy Blood and Cell Culture kit (Qiagen) or KAPA direct mouse genotyping kit (KAPA Biosystems). For each targeted locus, at least 3 sets of genotyping primers were designed, one set internal to detect the cassette, and two pairs spanning the junction of genomic sequences and targeting vector. Sequences of all primers are listed in Supplementary Table 8.

### Southern blot

External and internal probes were generated by PCR using the PCR DIG Probe Synthesis Kit (Roche). For the external probes we used HUES8 genomic DNA as the template and for internal probe generation the targeting plasmids. Genomic DNA was isolated from hPSCs by proteinase K digest and phenol/chloroform extraction. 10 ug of DNA was digested overnight with appropriate restriction enzymes, separated on a 0.8% agarose gel, denatured, neutralized and transferred by capillarity onto a Hybond-N membrane (GE Healthcare) using 10×SSC buffer. The hybridization was carried out overnight at 65 °C with rotation with a 5 ul of DIG labeled probe in 15 ml of hybridization buffer. Blots were prepared and washed using commercial buffers (DIG block and wash buffers, Roche) following manufacturer’s instructions. The DIG probe was detected with an AP-conjugated anti-DIG antibody followed by chemiluminescent detection (CDP-Star, Roche) and developed on film.

### Directed differentiation

To initiate pancreatic differentiation, the cells were dissociated using TrypLE Express to single cells and seeded at 150,000 cell/cm^2^ onto 1:30 dilution of growth factor reduced Matrigel (BD Biosciences) in DMEM/F12 in E8-MEF conditional media with 10 uM Y27632. Two days following seeding the differentiation was started. Day 1 cells were exposed to RPMI +3 uM CHIR-99021 (Stemgent) +100 ng/ml rhActivinA (R&D Systems). Day 2–3: +100 ng/ml rhActivinA. Day 4–5: +50 ng/ml FGF7 (Peprotech). Day 6–9: DMEM +50 ng/ml FGF7 + 2 μM RA (Sigma) +0.25 μM SANT-1 (Sigma) +100 ng/ml rhNoggin (R&D Systems).

To generate neural progenitor cells, H1 hPSCs were cultured on Matrigel coated plates in E8 media for two days before induction to the neural program for five days DMEM/F12:Neurobasal media at 1:1 ratio +1xB27 + 1×N2 + 2mM Glutamax (all Invitrogen).

Prior to cardiac differentiation, hPSCs were passaged onto Matrigel coated plates. At 70–95% confluence cells were exposed to rhActivinA and WNT3A for 1 day (R&D Systems) in Advanced RPMI (Invitrogen) supplemented with 2% KOSR (Gibco), ascorbic acid (Sigma), NEAA (Gibco), BSA (Gibco) and thioglycerol (Sigma). For the next 2 days, cells were treated with ActivinA, WNT3A, BMP4, and transferrin; at day 4 with BMP4 and transferrin; from day 6 onwards, with basal differentiation medium only.

### Antibody staining

For immunofluorescent analysis, cells were briefly washed with PBS and then fixed with 4% PFA in PBS for 20 min at RT followed by washing three times with PBST (PBS +0.1% Triton-X). The unspecific binging of antibodies was blocked by 30 min incubation with blocking solution (10% donkey serum in PBST) at RT. The primary antibodies were in blocking solution for 16 h at 4 °C with shaking and then cells were washed three times with PBST for 10 min. The secondary antibodies were conjugated with appropriated Alexa Fluor Dye (Jackson ImmunoResearch Laboratories), diluted with blocking solution and incubated with cells for 1 h at RT. Cells were then washed three times with PBST and nuclei were stained with DAPI (Invitrogen). All primary antibodies and dilutions are listed in Supplementary Table 10.

### Gene expression analysis

For quantitative RT-PCR, total RNA was isolated using Trizol (Invitrogen). The RNase-free DNAse treatment was used to remove any traces of genomic DNA according to the manufacturer’s protocol (Qiagen). One ug of RNA was used for reverse transcription using iScript (Biorad). 1/10 of cDNA was used for PCR using SYBR Green (KAPA Biosystems) and a Connect CFX light cycler (Biorad) (≤40 cycles). Primers were designed to span or amplify across the exon junctions using Primer3 software. The specificity of PCR products was verified by melt curve analysis using Precision Melt Analysis Software (Biorad) and gel electrophoresis, followed by TOPO cloning (Invitrogen) and Sanger sequencing. Threshold data were analyzed in CFX Manager Software v3.1 (Applied Biosystems) using the Comparative Ct relative quantitation method, with beta-actin and TBP as the endogenous controls. Sequences of primers are shown in Supplementary Table 9. All primers were purchased from IdtDNA.

### Statistical analysis

P values were calculated using t-test and PRISM6 software. For multiple comparison tests Bonferroni correction was used. Unless otherwise stated in figure legends, data are presented as mean ± SD and the following symbols are used to represent P values, *P < 0.05, **P < 0.01, ***P < 0.001, and ****P < 0.0001. N represents number of independent experiments.

## Additional Information

**How to cite this article**: Yang, D. *et al.* Enrichment of G2/M cell cycle phase in human pluripotent stem cells enhances HDR-mediated gene repair with customizable endonucleases. *Sci. Rep.* **6**, 21264; doi: 10.1038/srep21264 (2016).

## Supplementary Material

Supplementary Information

## Figures and Tables

**Table 1:** Cell cycle synchronization leads to a marked improvement in genome targeting by HDR regardless of cell line or gene target.

	Wnt5a	NeuroD1	Nkx6.1	Insulin	Oct4
Cells targeted	1. H1	1. H1	1. H1 2. HUES8	1. H1	1. FUCCI-H9
	2. NPCs	2. HUES8	3. DiPSCs	2. HUES8	
		3. DiPSCs	3. DiPSCs		
Targeting strategy	1. CRISPR nickase (D10A, *S. pyogenes*	1. CRISPR (*S. pyogenes*) 2. CRISPR (*N. meningitides*	1. ZFNs	1. CRISPR (*S. pyogenes*)2. TALENs	1. CRISPR (*S. pyogenes*
Average fold increase in efficiency with synchronization	3.75	4.66	5.32	4.61	5.86

Five genes are targeted in five different cell lines using three different targeting strategies, as listed. The effect of synchronization is listed as the difference in percent correct clones (synchronization improvement of efficiency) or the fold-increase in efficiency (fold increase in efficiency with synchronization).
