# Supplementary material for: Enrichment of G2/M cell cycle phase in human pluripotent stem cells enhances HDR-mediated gene repair with customizable endonucleases
Source: Sci Rep. 2016 Feb 18;6:21264. doi: 10.1038/srep21264 (PMC4757933; doi:10.1038/srep21264)
Supplement: Supplementary Information [file srep21264-s1.pdf]

Enrichment of G2/M cell cycle phase in human pluripotent stem cells enhances HDR-mediated gene repair with customizable endonucleases.

Diane Yang[1, 7], Marissa Scavuzzo[2, 7], Jolanta Chmielowiec[3, 4], Robert Sharp[3, 4], Aleksandar Bajic[6], Malgorzata Borowiak[1-5]*

[1] Molecular and Cellular Biology Department, Baylor College of Medicine, One Baylor Plaza, Houston, TX 77030, USA

[2] Program in Developmental Biology, Baylor College of Medicine, One Baylor Plaza, Houston, TX 77030, USA

[3] Stem Cells and Regenerative Medicine Center, Baylor College of Medicine, One Baylor Plaza, Houston, TX 77030, USA

[4] Center for Cell and Gene Therapy, Baylor College of Medicine, Texas Children's Hospital and Houston Methodist Hospital, Houston, TX 77030, USA

[5] McNair Medical Institute, Houston, TX 77030, USA

[6] Jan and Dan Duncan Neurological Research Institute, Texas Children's Hospital, 1250 Moursund Street, Houston, TX 77030

[7] equal contribution

* Corresponding author: borowiak@bcm.edu, phone +1 713 798 7771

Running title: Enhancing gene targeting in human PSCs by cell cycle manipulation

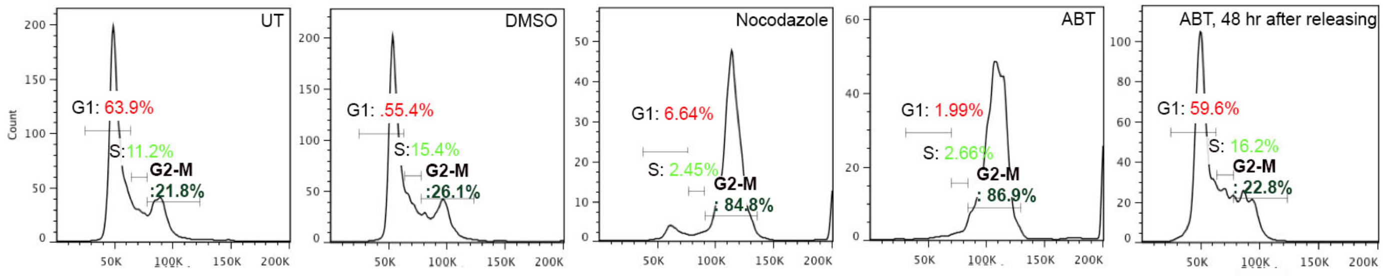

	UT	DMSO	Nocodazole	ABT
G1 (%)	59.13±4.88	63.83±9.08	4.27±1.89	4.37±3.11
S (%)	11.67±1.15	10.87±1.97	1.6±0.36	1.57±0.35
G2/M (%)	28.8±4.45	24.7±8.22	90.73±1.56	91.43±3.29

Supplementary Figure 1: Synchronized Fucci H9 cells detected measured by DNA content and flow cytometry. Representative FACS plots of synchronized Fucci H9 cells stained with Hoechst show efficient G2/M arrest in ABT or Nocodazole treated cells as compared to untreated and DMSO vehicle control cells (Top panel). ABT treatment shows a reversible effect of cell cycle after 48 hours of releasing. Lower panel shows Mean ± standard deviation for percentage of each cell cycle phase (*n*=3 biological replicates).

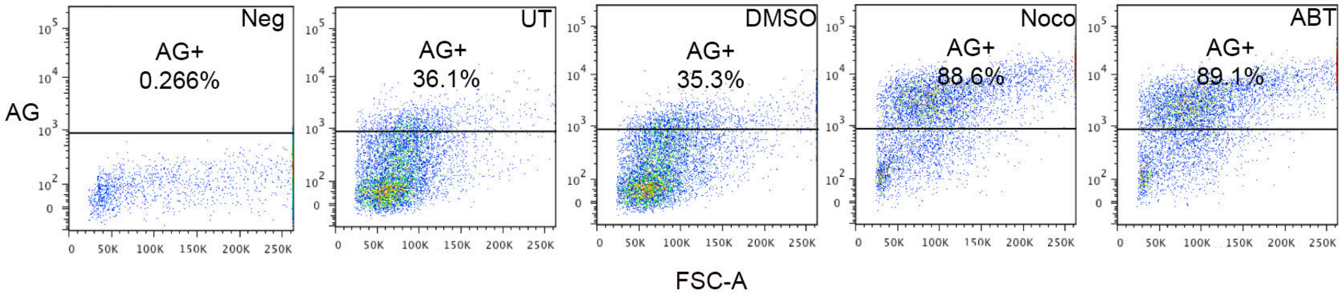

	UT	DMSO	Nocodazole	ABT
DN (%)	45.07±27.43	59.93±1.01	7.33±0.67	7.4±0.61
KO+(%)	9.4±15.07	0.67±0.06	3.37±0.67	2.03±1.59
AG+(%)	38.23±8.41	34.4±1.31	88.43±0.38	88.6±0.7

Supplementary Figure 2: G2/M cell cycle phase of Fucci H9 cells measured by AG fluorescence with and without synchronization using flow cytometry. Representative FACS plots of synchronized Fucci H9 cells show efficient G2/M arrest by ABT or Nocodazole treated cells as compared to untreated and DMSO vehicle control cells (Top panel). Lower panel shows Mean ±

standard deviation for percentage of each cell cycle phase (*n*=3 biological replicates). DN, double negative. KO, Kusabira orange. AG, Azami Green.

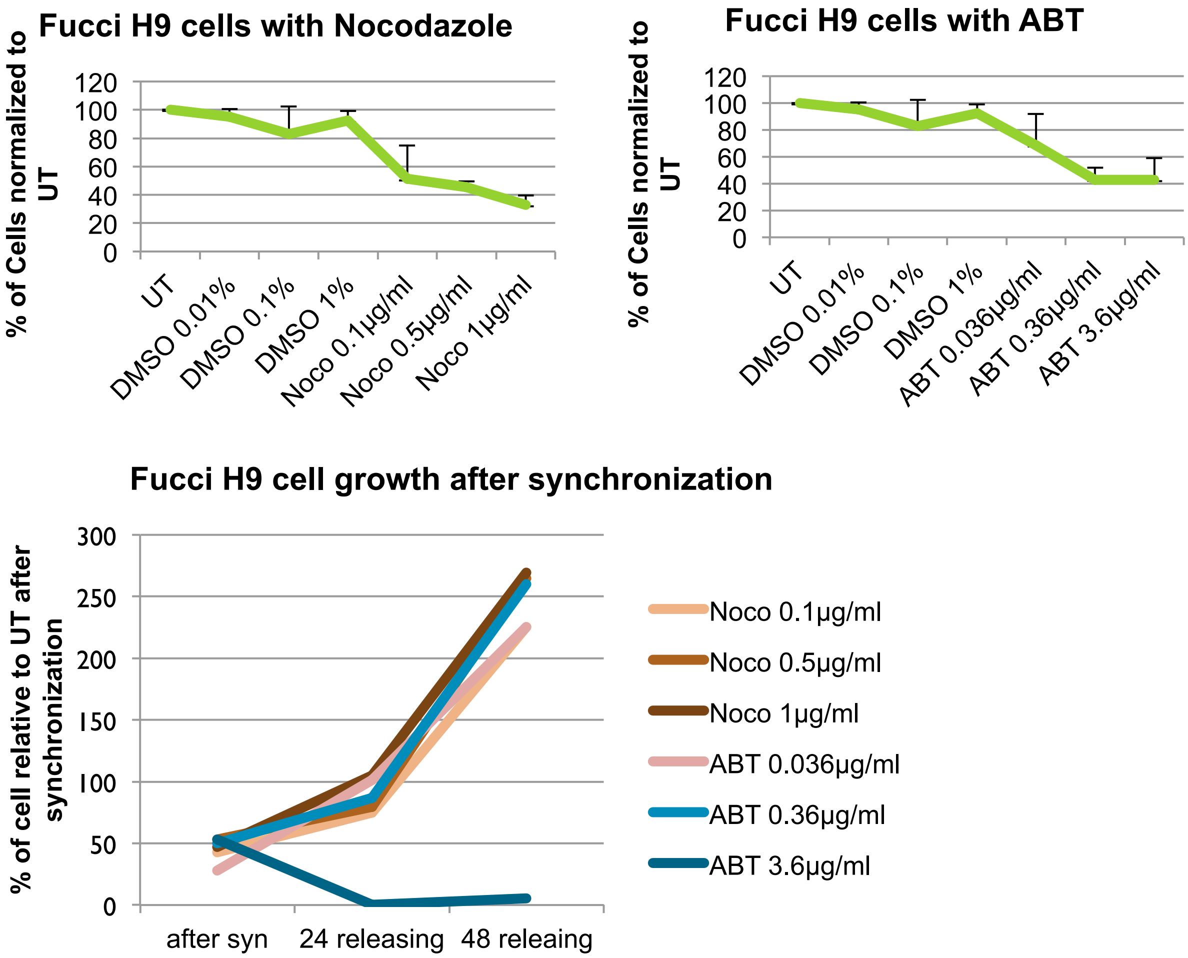

Supplementary Figure 3: Cell survival rate after synchronization with Nocodazole and ABT. Fucci H9 cell counts immediately after synchronization normalized to untreated cells show a decrease after treatment (Top panel, *n*=3 biological replicates). Nocodazole and ABT induce reversible growth arrest and cells recover their number around 30hrs post synchronization as shown by survival and recover cure normalized to untreated cell number at 0 hours post synchronization (Lower panel). The high concentration of ABT (3.6 μg/ml) inhibits cell number recovery.

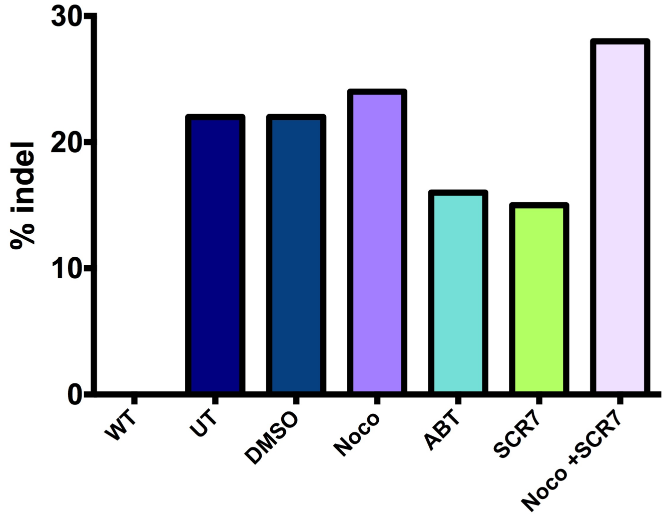

Supplementary Figure 4: Quantification of indel formation after targeting of Oct4 using CRISPR/Cas9 in HEK293t cells. CRISPR/Cas9 targeted region was amplified and indels were analyzed by T7 endonuclease assay in HEK293t untreated, DMSO vehicle treated, synchronized, and control untargeted cells.

Normalized to UT		BRCA1	BRCA2	RAD51	RAD52	LIG4	XRCC4	XRCC5	XRCC6
3hr	UT	1.00	1.00	1.00	1.00	1.000	1.00	1.00	1.00
	Noco	2.01	29.70	10.43	93.11	0.274	0.14	0.51	0.61
	ABT	0.22	0.74	0.27	1.61	1.407	1.34	1.00	1.38
6hr	UT	1.00	1.00	1.00	1.00	1.001	1.00	1.00	1.00
	Noco	1.28	0.77	1.51	7.55	1.360	0.66	0.58	0.70
	ABT	1.58	0.56	0.45	0.83	1.461	0.66	0.56	0.64
12hr	UT	1.00	1.00			1.000	1.00	1.00	1.00
	Noco	0.50	2.11	1.53		0.483	0.68	0.57	0.76
	ABT	2.43	3.25	2.62		1.665	1.06	0.53	0.49
24hr	UT	1.00	1.00	1.00	1.00	1.000	1.00	1.00	1.00
	Noco	1.33	1.52	2.00	13.20	3.305	0.79	1.04	1.41
	ABT	5.86	1.48	0.43	9.71	2.424	1.02	1.15	0.49

Low High

Supplementary Figure 5: HR related (*BRCA1, BRCA2, RAD51, RAD52*) and NHEJ related (*LIG4 XRCC4, XRCC5* and *XRCC6*) gene expression detected by qPCR. H1 synchronized cell gene expression analyzed by qPCR at 3, 6, 12, and 24 hours post nucleofection normalized to untreated cells (*n*=2 technical replicates).

Treatment	G0/G1 (%)	S (%)	G2/M (%)
UT	46.73±5.66	21.4±6.95	30.57±4.92
DMSO 1%	45.2±7.13	18±0.79	35.97±6.74
DMSO 0.1%	47.77±6.56	18.9±0.7	32.67±6.82
ABT 3.6μg/ml	5.87±2.57	16.93±6.45	76.6±9.4

ABT 0.36μg/ml	6.5±4.16	15.97±8.08	77±12.99
Noco 1μg/ml	5.07±2.23	13.5±2.65	81.43±5.25
Noco 0.5μg/ml	5.77±4.41	16.5±9.23	76.77±14.01

Supplementary Table 1- H1 hPSCs synchronized using different concentrations of Nocodazole and ABT (*n*=3 biological replicates). Data presented as Mean ± Standard deviation.

Treatment /Releasing time (hr)	Treatment	G1 (%)	S (%)	G2/M (%)
	UT	52±8.66	27.6±1.67	18.07±7.04
	DMSO	47.23±3.9	26.27±2.6	25.9±4.84
4/3	Nocodazole	25.53±1.1	26.83±1.15	48.83±1.81
	ABT	24.37±1.63	27.47±1.35	46.57±0.21
4/1	Nocodazole	33.63±1.79	23.47±1.98	41.36±0.35
	ABT	32.27±3.26	25.23±2.63	40.7±2.67
4/0	Nocodazole	34.6±2.05	23.2±0.87	40.13±2.05
	ABT	38.07±0.86	22.8±0.72	36.73±1.18
16/3	Nocodazole	23.9±15.25	6.41±1.36	68.37±17.55
	ABT	30.77±26.72	5.05±2.21	62.47±25.61
**16/1**	**Nocodazole**	**5.7±1.18**	**2.17±0.46**	**91±1.25**
	ABT	14.76±9.87	3.55±2.36	80.13±11.18
**16/0**	Nocodazole	6.41±1.59	2.77±0.59	89.8±2.88
	**ABT**	**6.51±3.2**	**2.86±0.99**	**89.23±4.42**
12/3	Nocodazole	11.64±8.83	2.75±0.66	84.43±9.79
	ABT	21.82±24.49	2.32±0.66	74.73±25.69
12/1	Nocodazole	5.54±0.23	2.6±1.3	61.72±48.61
	ABT	10.93±6.5	2.74±0.58	84.77±7.62
12/0	Nocodazole	8.34±2.41	3.27±1.58	84.53±2.54
	ABT	6.23±2.94	3.23±1.38	89.13±4.52
18/3	Nocodazole	9.7± 6.29	3.4±1	86.1±7.31
	ABT	14.13±5.23	5.72±2.45	79.47±7.48
18/1	Nocodazole	5.95±2.19	1.43±0.27	91.87±2.57
	ABT	5.95±1.35	1.6±0.48	91.47±1.52
18/0	Nocodazole	6.83±3.85	2.04±0.82	90.37±4.7
	ABT	8.25±3.01	1.74±0.23	89.27±2.68

Supplementary Table 2- H1 hPSCs with different synchronization and releasing time. Treatment and releasing time are annotated as Treatment/Releasing time (hr) (*n*=3 biological replicates). Data presented as Mean ± Standard deviation.

Program	GFP+(%)	Viability
CA137	67.6	~60%
CB150	37.4	~60%
CD118	29.2	~80%
CE118	28.6	~80%
CM113	38.6	~80%
DC100	66.3	~30%
DN100	84.4	~20%

Supplementary Table 3: Various nucleofection programs were tested using 10ug pMAXGFP of 2.5*10^5 of H1. GFP and cell viability was measured by FACS after 48 hours of nucleofection.

**Gene target**	**Targeting method**	**Plasmid**
WNT5A	CRISPR; D10A Cas9; *S. pyogenes*	Targeting: pX335<br>Donor(s): PL452
NEUROD1 1-3	CRISPR; Cas9; *S. pyogenes*	Targeting: pX459<br>Donor(s): OCT4-2A-eGFP-PGK-Puro; Kume_mKO1_pPL452loxP_EM7_PGK_neo
NEUROD1 4-5	CRISPR; Cas9; *N. meningitides*	Targeting: pSimpleII-U6-tracr-U6-BsmBI-NLS-NmCas9-HA-NLS<br>Donor(s): OCT4-2A-eGFP-PGK-Puro; Kume_mKO1_pPL452loxP_EM7_PGK_neo
NKX6.1	ZFNs	Targeting: PZFN1_Nkx6.1 and PZFN2_Nkx6.1<br>Donor(s): OCT4-2A-eGFP-PGK-Puro
INS	CRISPR; Cas9 TALENs	Targeting: 13ABPZVC_INS_TAL_3R_TALtrunc_FokI<br>Donor(s): Kume_mKO1_pPL452loxP_EM7_PGK_neo
OCT4	CRISPR; Cas9; *S. pyogenes*	Targeting: pX330<br>Donor(s): CMV-Brainbow-1.1M and Oct4-eGFP-PGK-Puro

Supplementary Table 4: Plasmids for gene targeting method and donor plasmids for HDR. *NEUROD1* CRISPR/Cas9 are separated by species with sgRNA numbers corresponding to Supplementary Table 5.

**Gene target**	**Targeting strategy**	**Guide set**	**Direction**	**Sequence (5' to 3')**
	CRISPR nickase	1	F	CACCGTGCAGTTCCACCTTCGATGTCGG
	CRISPR nickase	1	R	AAACCCCGACATCGAAGGTGGAACTGCAC

WNT5A	CRISPR nickase	2	F	CACCGTTTGGCAGGGTGATGCAGATAGG
	CRISPR nickase	2	R	AAACCCTATCTGCATCACCCTGCCAAAC
	CRISPR nickase	3	F	CACCGTTCGATGTCGGAATTGATACTGG
	CRISPR nickase	3	R	AAACCCAGTATCAATTCCGACATCGAAC
	CRISPR nickase	4	F	CACCGGTGGATAACACCTCTGTTTTTGG
	CRISPR nickase	4	R	AAACCCAAAAACAGAGGTGTTATCCACC
NEUROD1	CRISPR	1	F	CACCGAGCCACGGATCAATCTTCTC
	CRISPR	1	R	AAACGAGAAGATTGATCCGTGGTCC
	CRISPR	2	F	CACCGGCACTATCCTGCAGCGACAC
	CRISPR	2	R	AAACGTGTCGCTGCAGGATAGTGCC
	CRISPR	3	F	CACCGGCACTATCCTGCAGCGACAC
	CRISPR	3	R	AAACGTGTGGCTGCAGGATAGTGCC
	CRISPR	4	F	GAGCCCTTCTTTGAAAGCCCTCT
	CRISPR	4	R	AGAGGGCTTTCAAAGAAGGGCTC
	CRISPR	5	F	AGTGCCCAGCTCAATGCCATATT
	CRISPR	5	R	AATATGGCATTGAGCTGGGCACT
NKX6.1	ZFN	1	-	CTCTACTTCAGCCCCAGnnCCGCGGCCGTGGCCGCCGG
	ZFN	2	-	GGACGCCCATCTTCTGGnCCGGAGTGATGCAGAGCC
	ZFN	3	-	**CTGGCCGGACGCCCATCTTCnGGCCCGGAGTGATGCAGAGCC**
	ZFN	4	-	CCGGTACCCCAAGCCGCnnGCTGAGCTGCCTGGCCGGC
	ZFN	5	-	CGGACGCCCATCTTCTGnnCCGGAGTGATGCAGAGCC
	ZFN	6	-	TACTTCAGCCCCAGCGCCGCnnCCGTGGCCGCCGTGGGCCGG
	ZFN	7	-	CTGGCCGGACGCCCATCTTCTnGCCCGGAGTGATGCA
	ZFN	8	-	CGCCGTGGGCCGGTACCCCAAnnCGCTGGCTGAGCTG
	ZFN	9	-	CCGGTACCCCAAGCCGCTGGnnGAGCTGCCTGGCCGGC
	ZFN	10	-	CGTGGGCCGGTACCCCAAGCnnCTGGCTGAGCTGCCTG
	ZFN	11	-	GGCCGCCGTGGGCCGGTACCnnAAGCCGCTGGCTGAGT

	ZFN	12	-	CCTGGCCGGACGCCCATCTTnT GGCCCGGAGTGATGCAGAG
	ZFN	13	-	GGCCGCCGTGGGCCGGTACCCn AAGCCGCTGGCTGAGT
	ZFN	14	-	GACGCCCATCTTCTGGCCCGnn GTGATGCAGAGCCCGCC
	ZFN	15	-	CTGGCCGGACGCCCATCnnCTG GCCCGGAGTGATGCAG
	ZFN	16	-	CGGACGCCCATCTTCTGGCCnn GAGTGATGCAGAGCCCGCCCT
INS	TALENs	1	F	TCTGCTCCCTCTACCAGCT
	TALENs	1	R	TAGACGCAGCCCGCAGGCA
OCT4	CRISPR	1	F	CACCGCTCTCCCATGCATTCAAA CTGAGG
	CRISPR	1	R	AAACCCTCAGTTTGAATGCATGG GAGAGC

Supplementary Table 5: Guide sequences for gene targeting.

**Gene target**	**Chromosomal location**	**Sequence**
WNT5A first constitutive exon	chr3:55,513,341-55,513,466	atgcagtacatcggagaaggcgcgaagacag gcatcaaagaatgccagtatcaattccgacatcg aaggtggaactgcagcactgtggataacacctct gtttttggcagggtgatgcagataggcagccgcg agacggccttcacatacgcggtgagcgcagca ggggtggtgaacgccatgagccgggcgtgccg cgagggcgagctgtccacctgcggctg
NEUROD1, exon 1	chr2:182,540,833-182,545,392	**acgcctacagcgcagcgctggagcccttctt tgaaagccctctgactgattgcaccagccctt cctttgatggacccctcagcccgccgctcag catcaatggcaacttctctttcaaacacgaac cgtccgccgagtttgagaaaattatgccttta ccatgcactatcctgcagcgacactggcagg ggcccaaagccacggatcaatcttctcaggc accgctgcccctcgctgcgagatccccatag acaatattatgtccttcgatagccattcacatc atgagcgagtcatgagtgcccagctcaatgc catatttcatgattag**
NKX6.1, 3' of exon 1	chr4:85,403,140-85,429,603	gcggccgtggccgccgtgggccggtaccccaa gccgctggctgagctgcctggtggccggacgcc catcttctggcccggagtgatgcagagcccgccc tggagggacgcacgcctggcctgtacccctcgtg agtac
INS	chr11:2,171,009-2,192,571	tggggcaggtggagctgggcgggggccctggt gcaggcagcctgcagcccttggccctggaggg gtccctgcagaagcgtggcattgtggaacaatgc tgtaccagcatctgctccctctaccagctggagaa ctactgcaactagacgcagcccgcaggcagcc ccacacccgccgcctcctgcaccgagagagat ggaataaagcccttgaaccagc

OCT4	chr6_ssto_hap7:2,467,051-2,473,404	cctcggtccctttccctgaggggaagcctttccc<br>cctgtctctgtcaccactctgggctctcccatgcatt<br>caaactgaggtgcctgcccttctaggaatggggg<br>acaggggagggaggagctagggaaag

Supplementary Table 6: Genomic location of targeting. Red sequence shows complementary sequence binding targeting guides.

Homologous arms of donor	Sequence	Mutated sgRNA binding site
WNT5a left homology arm, guides 1 & 2; 3 & 4	CGTCATGGTACCCCTCACAATCACATAAGGA<br>GCCCTTTTAAAATGTCAAGAAACATGGTGAAT<br>TTTCTCCAGTCTGTATACTTGTCCCTCTGTTC<br>TTCCATCAGTCAAAAGGAACTGCTATCTCCTA<br>CCCGTATCTAATTGTGTCTACTTTCAGCATTC<br>ATAAGTTTTCAAGGCAGATTGGTCCAATTTGT<br>GGGGCAAAGTGGGTCACTTTCTCTAATGTCA<br>GAGGCCTGTCCCTCAAACTGCTGTTTCCTCC<br>TTTTCTGAGTGGATTTTGGAAATACTGGTTCA<br>GGTATATGGTGACAGGGGCTAGGAAGGGAG<br>TGAAAATACTAACTGAGGAGCCAGATGGCAG<br>GATGGGGACTGCACAATACCTCCGTTTTCTG<br>AGTGCAGTTTAAACAGAGGAGTTTTATGAAG<br>CCCTTCTCATACTTTGTCATGAGGACAAGCA<br>GGAGAGAAAAAAGCATCTGTGTGCTTAAAAC<br>TATGTATTGCTGACATGCTTTCAGGGGGCCC<br>ACCTCGAGATTTCACGTAAATCTTGAATGCAC<br>ATCTCCCCCCCTTGCAGGTCGCTAGGTATGA<br>ATAACCCTGTTCAGATGTCAGAAGTATATATT<br>ATAGGAGCACAGCCTCTCTGCAGCCAACTGG<br>CAGGACTTTCTCAAGGACAGAAGAAACTGTG<br>CCACTTGTATCAGGACCACATGCAGTACATC<br>GGAGAAGGCGCGAAGACAGGCATCAAAGAA<br>TGAATGTTTCAATTATCACATCCTTGGTGGAA<br>CTGCAGCAGTCGACCTAGCTG	ATCACATC<br>CTTGGTGG<br>AACTGCA
WNT5a right homology arm, guides 1 & 2; 3 & 4	GTCAGTAAGCGGCCGCCTGTGGATAACACC<br>GTGGTTTTGAACAGGGTGATGCTTATTAATAG<br>GAAAACATTTAAAATATTTTTTTAAAACATTAA<br>CTTTTTTTCCTAGAAGAAATCCTTAGCATTTAC<br>TTCATGCTTGATAGAAAGCATGAAGGAGGAT<br>GCCTTCCAGCTCGGGTGGTGGCTTGGGCATT<br>TTTGTCATTATAGCAAATGATGTTAATTCCTGT<br>CCCTACACCTGATGATAAGTTTTGGATTTTAA<br>TCAAATCAGGAGGCAAGTATCACAGACACCT<br>TCATTTTTGCAAACACTTAATTTTTCTATAAAG<br>CATTTTGTTTAAAATAGAAGCAGAACACTTAT<br>TTTGGTTTCTTAAGAGTAGGTGGGGGAAGCA<br>AGTCATGTATATGCTTTTAAACGTCGAAACTC<br>TCTTGCCTGTTAGTTTTTTAAGCCACAATTGA<br>AACGGCCCGAGCATCGAACTAAAAGTTGCCG	TTGAACAG<br>GGTGATGC<br>TTATTAA

	TCTTTTAGAATCCTGGATATATTGAAAGTGAA<br>CAAAGTTTTAATTTTCCTAATAGAACTATTTCC<br>TGAGGCTATAAAGATATAGACGTTAAAAGCTT<br>TATTTTTAGTCCCAGAAAGAATTTTAATTAAAA<br>GTGATCCAATTTATGTTTGTGATGTCAGCAAT<br>TGATATAATAAAAGCACATGCTTGGGATGTCA<br>ACCTAGGAACTTGAAAAAAAAATTAAGAGTAT<br>TGCTCACAGTCCGATTCTTCATTTTAAATACG<br>TTGTTGAATGTCTTACTTCCCCATTCAAAGAG<br>ATAGCATTTCAAATGGGCTAAAGGCAAAGATT<br>TTAAAAAATCTAGTAATGATAGTGAGATCCAG<br>ACATGATAAGATACATTGATGAGTTTGGACAA<br>ACCACAACTAGAATGCAGTGAAAAAAATGCTT<br>TATTTGTGAAATTTGTGATGCTATTGCTTTATT<br>TGTAACCATTATAAGCTGCAATAAACAAGTTC<br>CGCGGCTATGAC	
NEUROD1 left homology arm, guide 1	TTAATTggatccATGAAGGCTAACGCCCGGGAG<br>CGGAACCGCATGCACGGACTGAACGCGGCG<br>CTAGACAACCTGCGCAAGGTGGTGCCTTGCT<br>ATTCTAAGACGCAGAAGCTGTCCAAAATCGA<br>GACTCTGCGCTTGGCCAAGAACTACATCTGG<br>GCTCTGTCGGAGATCCTGCGCTCAGGCAAAA<br>GCCCAGACCTGGTCTCCTTCGTTCAGACGCT<br>TTGCAAGGGCTTATCCCAACCCACCACCAAC<br>CTGGTTGCGGGCTGCCTGCAACTCAATCCTC<br>GGACTTTTCTGCCTGAGCAGAACCAGGACAT<br>GCCCCCCCACCTGCCGACGGCCAGCGCTTC<br>CTTCCCTGTACACCCCTACTCCTACCAGTCG<br>CCTGGGCTGCCCAGTCCGCCTTACGGTACCA<br>TGGACAGCTCCCATGTCTTCCACGTTAAGCC<br>TCCGCCGCACGCCTACAGCGCAGCGCTGGA<br>GCCCTTCTTTGAAAGCCCTCTGACTGATTGC<br>ACCAGCCCTTCCTTTGATGGACCCCTCAGCC<br>CGCCGCTCAGCATCAATGGCAACTTCTCTTT<br>CAAACACGAACCGTCCGCCGAGTTTGAGAAA<br>AATTATGCCTTTACCATGCACTATCCTGCAGC<br>GACACTGGCAGGGGCCCAAAGCCACGGATC<br>AATCTTtgctagcTTAATT	AGCCACGG<br>ATCAATCTT<br>tgc
NEUROD1 right homology arm, guide 1	TTAATTggcgcgccCTCAGGCACCGCTGCCCCT<br>CGCTGCGAGATCCCCATAGACAATATTATGT<br>CCTTCGATAGCCATTCACATCATGAGCGAGT<br>CATGAGTGCCCAGCTCAATGCCATATTTCAT<br>GATTAGAGGCACGCCAGTTTCACCATTTCCG<br>GGAAACGAACCCACTGTGCTTACAGTGACTG<br>TCGTGTTTACAAAAGGCAGCCCTTTGGGTAC<br>TACTGCTGCAAAGTGCAAATACTCCAAGCTTC<br>AAGTGATATATGTATTTATTGTCATTACTGCCT<br>TTGGAAGAAACAGGGGATCAAAGTTCCTGTT<br>CACCTTATGTATTATTTTCTATAGCTCTTCTAT<br>TTAAAAAATAAAAAAATACAGTAAAGTTTAAAA<br>AATACACCACGAATTTGGTGTGGCTGTATTCA<br>GATCGTATTAATTATCTGATCGGGATAACAAA	ttaattggcgcg<br>ccCTC

	ATCACAAGCAATAATTAGGATCTATGCAATTTTTAAACTAGTAATGGGCCAATTAAAATATATATAAATATATATTTTTCAACCAGCATTTTACTACTTGTTACCTTTCCCATGCTGAATTATTTTGTTGTGATTTTGTACAGAATTTTTAATGACTTTTTATAATGTGGATTTCCTATTTTAAAACCATGCAGCTTCATCAATTTTTATACATATCgcggccgcTTAATT	
NEUROD1 left homology arm, guide 2	TTAATTggatccACTAAGGCTCGCCTGGAGCGTTTTAAATTGAGACGCATGAAGGCTAACGCCCGGGAGCGGAACCGCATGCACGGACTGAACGCGGCGCTAGACAACCTGCGCAAGGTGGTGCCTTGCTATTCTAAGACGCAGAAGCTGTCCAAAATCGAGACTCTGCGCTTGGCCAAGAACTACATCTGGGCTCTGTCGGAGATCCTGCGCTCAGGCAAAAGCCCAGACCTGGTCTCCTTCGTTCAGACGCTTTGCAAGGGCTTATCCCAACCCACCACCAACCTGGTTGCGGGCTGCCTGCAACTCAATCCTCGGACTTTTCTGCCTGAGCAGAACCAGGACATGCCCCCCCACCTGCCGACGGCCAGCGCTTCCTTCCCTGTACACCCCTACTCCTACCAGTCGCCTGGGCTGCCCAGTCCGCCTTACGGTACCATGGACAGCTCCCATGTCTTCCACGTTAAGCCTCCGCCGCACGCCTACAGCGCAGCGCTGGAGCCCTTCTTTGAAAGCCCTCTGACTGATTGCACCAGCCCTTCCTTTGATGGACCCCTCAGCCCGCCGCTCAGCATCAATGGCAACTTCTCTTTCAAACACGAACCGTCCGCCGAGTTTGAGAAAAATTATGCCTTTACCATGCACTATCCTGCAGCGAtggctagcTTAATT	GCACTATCCTGCAGCGAtgg
NEUROD1 right homology arm, guide 2	TTAATTggcgcgccCACTGGCAGGGGCCCAAAGCCACGGATCAATCTTCTCAGGCACCGCTGCCCCTCGCTGCGAGATCCCCATAGACAATATTATGTCCTTCGATAGCCATTCACATCATGAGCGAGTCATGAGTGCCCAGCTCAATGCCATATTTCATGATTAGAGGCACGCCAGTTTCACCATTTCCGGGAAACGAACCCACTGTGCTTACAGTGACTGTCGTGTTTACAAAAGGCAGCCCTTTGGGTACTACTGCTGCAAAGTGCAAATACTCCAAGCTTCAAGTGATATATGTATTTATTGTCATTACTGCCTTTGGAAGAAACAGGGGATCAAAGTTCCTGTTCACCTTATGTATTATTTTCTATAGCTCTTCTATTTAAAAAATAAAAAAATACAGTAAAGTTTAAAAAATACACCACGAATTTGGTGTGGCTGTATTCAGATCGTATTAATTATCTGATCGGGATAACAAAATCACAAGCAATAATTAGGATCTATGCAATTTTTAAACTAGTAATGGGCCAATTAAAATATATATAAATATATATTTTTCAACCAGCATTTTACTACTTGTTACCTTTCCCATGCTGAATTATTTTGTTGTGATTTTGTACAGAATTTTTAATGACTTTTTATAATGTGGATTTCCTATTTTAgcggccgcTTAATT	ttaattggcgcgccCAC

NEUROD1 left homology arm, guide 3	TTAATTggatccGATGAGGACCTGGAAGAGGAGGAAGAAGAGGAAGAGGAGGATGACGATCAAAAGCCCAAGAGACGCGGCCCCAAAAAGAAGAAGATGACTAAGGCTCGCCTGGAGCGTTTTAAATTGAGACGCATGAAGGCTAACGCCCGGGAGCGGAACCGCATGCACGGACTGAACGCGGCGCTAGACAACCTGCGCAAGGTGGTGCCTTGCTATTCTAAGACGCAGAAGCTGTCCAAAATCGAGACTCTGCGCTTGGCCAAGAACTACATCTGGGCTCTGTCGGAGATCCTGCGCTCAGGCAAAAGCCCAGACCTGGTCTCCTTCGTTCAGACGCTTTGCAAGGGCTTATCCCAACCCACCACCAACCTGGTTGCGGGCTGCCTGCAACTCAATCCTCGGACTTTTCTGCCTGAGCAGAACCAGGACATGCCCCCCCACCTGCCGACGGCCAGCGCTTCCTTCCCTGTACACCCCTACTCCTACCAGTCGCCTGGGCTGCCCAGTCCGCCTTACGGTACCATGGACAGCTCCCATGTCTTCCACGTTAAGCCTCCGCCGCACGCCTACAGCGCAGCGCTGGAGCCCTTCTTTGAAAGCCCTCTGACTGATTGCACCAGCCCTTCCTTTGATGGACCCCTCAGCCCGCCGCTCAGCATggctagcTTAATT	AGCCCGCCGCTCAGCATggc
NEUROD1 right homology arm, guide 3	TTAATTggcgcgccCAATGGCAACTTCTCTTTCAAACACGAACCGTCCGCCGAGTTTGAGAAAAATTATGCCTTTACCATGCACTATCCTGCAGCGACACTGGCAGGGGCCCAAAGCCACGGATCAATCTTCTCAGGCACCGCTGCCCCTCGCTGCGAGATCCCCATAGACAATATTATGTCCTTCGATAGCCATTCACATCATGAGCGAGTCATGAGTGCCCAGCTCAATGCCATATTTCATGATTAGAGGCACGCCAGTTTCACCATTTCCGGGAAACGAACCCACTGTGCTTACAGTGACTGTCGTGTTTACAAAAGGCAGCCCTTTGGGTACTACTGCTGCAAAGTGCAAATACTCCAAGCTTCAAGTGATATATGTATTTATTGTCATTACTGCCTTTGGAAGAAACAGGGGATCAAAGTTCCTGTTCACCTTATGTATTATTTTCTATAGCTCTTCTATTTAAAAAATAAAAAAATACAGTAAAGTTTAAAAAATACACCACGAATTTGGTGTGGCTGTATTCAGATCGTATTAATTATCTGATCGGGATAACAAAATCACAAGCAATAATTAGGATCTATGCAATTTTTAAACTAGTAATGGGCCAATTAAAATATATATAAATATATATTTTTCAACCAGCATTTTACTACTTGgcggccgcTTAATT	ttaattggcgcgccCAA
NEUROD1 left homology arm, guide 4	TTAATTggatccGAGGACGACCTCGAAACCATGAACGCAGAGGAGGACTCACTGAGGAACGGGGGAGAGGAGGAGGACGAAGATGAGGACCTGGAAGAGGAGGAAGAAGAGGAAGAGGAGGATGACGATCAAAAGCCCAAGAGACGCGGCCCCAAAAAGAAGAAGATGACTAAGGCTCGCCTGGAGCGTTTTAAATTGAGACGCATGAAGGCTAACGCCCGGGAGCGGAACCGCATGCACGGACT	GAGCCCTTCTTTGAAAGCCCggc

	GAACGCGGCGCTAGACAACCTGCGCAAGGT<br>GGTGCCTTGCTATTCTAAGACGCAGAAGCTG<br>TCCAAAATCGAGACTCTGCGCTTGGCCAAGA<br>ACTACATCTGGGCTCTGTCGGAGATCCTGCG<br>CTCAGGCAAAAGCCCAGACCTGGTCTCCTTC<br>GTTCAGACGCTTTGCAAGGGCTTATCCCAAC<br>CCACCACCAACCTGGTTGCGGGCTGCCTGC<br>AACTCAATCCTCGGACTTTTCTGCCTGAGCA<br>GAACCAGGACATGCCCCCCCACCTGCCGAC<br>GGCCAGCGCTTCCTTCCCTGTACACCCCTAC<br>TCCTACCAGTCGCCTGGGCTGCCCAGTCCG<br>CCTTACGGTACCATGGACAGCTCCCATGTCT<br>TCCACGTTAAGCCTCCGCCGCACGCCTACAG<br>CGCAGCGCTGGAGCCCTTCTTTGAAAGCCCg<br>gctagcTTAATT	
NEUROD1 right homology arm, guide 4	TTAATTggcgcgccTCTGACTGATTGCACCAGCC<br>CTTCCTTTGATGGACCCCTCAGCCCGCCGCT<br>CAGCATCAATGGCAACTTCTCTTTCAAACACG<br>AACCGTCCGCCGAGTTTGAGAAAAATTATGC<br>CTTTACCATGCACTATCCTGCAGCGACACTG<br>GCAGGGGCCCAAAGCCACGGATCAATCTTCT<br>CAGGCACCGCTGCCCCTCGCTGCGAGATCC<br>CCATAGACAATATTATGTCCTTCGATAGCCAT<br>TCACATCATGAGCGAGTCATGAGTGCCCAGC<br>TCAATGCCATATTTCATGATTAGAGGCACGCC<br>AGTTTCACCATTTCCGGGAAACGAACCCACT<br>GTGCTTACAGTGACTGTCGTGTTTACAAAAG<br>GCAGCCCTTTGGGTACTACTGCTGCAAAGTG<br>CAAATACTCCAAGCTTCAAGTGATATATGTAT<br>TTATTGTCATTACTGCCTTTGGAAGAAACAGG<br>GGATCAAAGTTCCTGTTCACCTTATGTATTAT<br>TTTCTATAGCTCTTCTATTTAAAAAATAAAAAA<br>ATACAGTAAAGTTTAAAAAATACACCACGAAT<br>TTGGTGTGGCTGTATTCAGATCGTATTAATTA<br>TCTGATCGGGATAACAAAATCACAAGCAATAA<br>TTAGGATCTATGCAATTTTTAAACTAGTAATG<br>GGCCAATTAAAATgcggccgcTTAATT	ttaattggcgcg<br>ccTCT
NEUROD1 left homology arm, guide 5	TTAATTggatccAGACGCAGAAGCTGTCCAAAA<br>TCGAGACTCTGCGCTTGGCCAAGAACTACAT<br>CTGGGCTCTGTCGGAGATCCTGCGCTCAGG<br>CAAAAGCCCAGACCTGGTCTCCTTCGTTCAG<br>ACGCTTTGCAAGGGCTTATCCCAACCCACCA<br>CCAACCTGGTTGCGGGCTGCCTGCAACTCAA<br>TCCTCGGACTTTTCTGCCTGAGCAGAACCAG<br>GACATGCCCCCCCACCTGCCGACGGCCAGC<br>GCTTCCTTCCCTGTACACCCCTACTCCTACCA<br>GTCGCCTGGGCTGCCCAGTCCGCCTTACGG<br>TACCATGGACAGCTCCCATGTCTTCCACGTT<br>AAGCCTCCGCCGCACGCCTACAGCGCAGCG<br>CTGGAGCCCTTCTTTGAAAGCCCTCTGACTG<br>ATTGCACCAGCCCTTCCTTTGATGGACCCCT<br>CAGCCCGCCGCTCAGCATCAATGGCAACTTC	AGTGCCCA<br>GCTCAATG<br>CCATagc

	TCTTTCAAACACGAACCGTCCGCCGAGTTTG<br>AGAAAAATTATGCCTTTACCATGCACTATCCT<br>GCAGCGACACTGGCAGGGGCCCAAAGCCAC<br>GGATCAATCTTCTCAGGCACCGCTGCCCCTC<br>GCTGCGAGATCCCCATAGACAATATTATGTC<br>CTTCGATAGCCATTCACATCATGAGCGAGTC<br>ATGAGTGCCCAGCTCAATGCCATagctagcTTA<br>ATT	
NEUROD1 right homology arm, guide 5	TTAATTggcgcgccATTTCATGATTAGAGGCACG<br>CCAGTTTCACCATTTCCGGGAAACGAACCCA<br>CTGTGCTTACAGTGACTGTCGTGTTTACAAAA<br>GGCAGCCCTTTGGGTACTACTGCTGCAAAGT<br>GCAAATACTCCAAGCTTCAAGTGATATATGTA<br>TTTATTGTCATTACTGCCTTTGGAAGAAACAG<br>GGGATCAAAGTTCCTGTTCACCTTATGTATTA<br>TTTTCTATAGCTCTTCTATTTAAAAAATAAAAA<br>AATACAGTAAAGTTTAAAAAATACACCACGAA<br>TTTGGTGTGGCTGTATTCAGATCGTATTAATT<br>ATCTGATCGGGATAACAAAATCACAAGCAATA<br>ATTAGGATCTATGCAATTTTTAAACTAGTAAT<br>GGGCCAATTAAAATATATATAAATATATATTTT<br>TCAACCAGCATTTTACTACTTGTTACCTTTCC<br>CATGCTGAATTATTTTGTTGTGATTTTGTACA<br>GAATTTTTAATGACTTTTTATAATGTGGATTTC<br>CTATTTTAAAACCATGCAGCTTCATCAATTTTT<br>ATACATATCAGAAAAGTAGAATTATATCTAATT<br>TATACAAAATAATTTAACTAATTTAAACCAGCA<br>GAAAAGTGCTTAGAAAGTTATTGTGTTGCCTT<br>AGCACTTCTTTCCTCTCCAATTGTAAAAAAAA<br>gcggccgcTTAATT	ttaattggcgcg<br>ccATT
NKX6.1, left homology arm, ZFN 3	AGCCAGGGATCGAATCTAGGACTCGCGGAA<br>CGAAAGGACTGCCTAGCCCGCCGGGACGCC<br>TGCTTTTCTCGGCGAGCTGCCGCCTCCCGCG<br>TGGAGGGTTTGGACATCTCTGCTGCGCAGCT<br>AGGCGAGCAACTCCCGGCAGCGGCATTTTTG<br>GTTCAGTTGGCAGCTCGCCTCCGGGCGCGC<br>CGAGTGCCTCTCCGCTCGCGCCCTCGGCGC<br>TTCCGGCTCCTCTGAGCCCCGCGGGGGGCA<br>CCAGCCAGCGCCCTCGCTGCAAGGCTACGG<br>TCTCCGGCGTGGCCGTGGGATGTTAGCGGT<br>GGGGGCAATGGAGGGCACCCGGCAGAGCG<br>CATTCCTGCTCAGCAGCCCTCCCCTGGCCGC<br>CCTGCACAGCATGGCCGAGATGAAGACCCC<br>GCTGTACCCTGCCGCGTATCCCCCGCTGCCT<br>GCCGGCCCCCCCTCCTCCTCGTCCTCGTCGT<br>CGTCCTCCTCGTCGCCCTCCCCGCCTCTGG<br>GCACCCACAACCCAGGCGGCCTGAAGCCCC<br>CGGCCACGGGGGGGCTCTCATCCCTCGGCA<br>GCCCCCCGCAGCAGCTCTCGGCCGCCACCC<br>CACACGGCATCAACGATATCCTGAGCCGGCC<br>CTCCATGCCCGTGGCCTCGGGGGCCGCCCT<br>GCCCTCCGCCTCGCCCTCCGGTTCCTCCTCC	CTGGCCGG<br>ACGCCCAT<br>CTtctggCCC<br>GGAGTGAT<br>GCAGAGCC

	TCCTCTTCCTCGTCCGCCTCTGCCTCCTCCG CCTCTGCCGCCGCCGCGGCTGCTGCCGCGG CCGCAGCCGCCGCCTCATCCCCGGCGGGGC TGCTGGCCGGACTGCCACGCTTTAGCAGCCT GAGCCCGCCGCCGCCGCCGCCCGGGCTCTA CTTCAGCCCCAGCGCCGCGGCCGTGGCCGC CGTGGGCCGGTACCCCAAGCCGCTGGCTGA GCTGCCTGGCCGGACGCCCATCTtctggGCTA GC	
NKX6.1, right homology arm, ZFN 3	CCCGGAGTGATGCAGAGCCCGCCCTGGAGG GACGCACGCCTGGCCTGTACCCCTCGTGAG TACTACCACCCGCGCCCCGATGCCTGCCTGC CGTGCCTGTTCTGCCCACTCCCGGGTCGCG GCCCCTGGTGTGCATGCCGCTCAGTCCATCC TGTGGCCCGCCCCAGTAGTTTGACAGCGCC GGAATCACACCAGTTAGGTTGGGCCAGGGCT TCCAGAAAGGGATTCTCGCTTTTCTGAGCTC GCGTGGCTGTATCCAAGCGCCTCCCTCTCTC TAAGTCTTGAGTACGCCTGGGCTGAGCCGGT GTGTGTGTGTTCGTGTGGACCACAACGCGGT GAGGGACGTGTGTCTCGTCCGAGGCCCTGG CTGTTCCTGAAACGGCCTGGCCAGGACCGC AGGCCTGGCCTGGATTAGAAAGATGGGGAG GAGAGGGGAGGAGAGGCCACTGCATCTCTG CTTTCCCGGCTCCTCCCTGTCGCCAGCGCCC CATTCCGCCTTCGCCAGCCTAACCGCTGCCT CTCTTCGAAGCAGTGAGGCCCGGCAGCGGT GAGCCCTCCACCGCCCGGGAAGTGAGTGTA TTTGCATGCACGTCTCCGCCTGGGGTGCCCC ACCTCACCTCTTCGCCCTCGCCGCCCGAGTA CACTCGGCCTGGGTGAGCCCATTGAAGACA GTCCAGCGTCCCCAGGGCAGGGCGAGCGCT TCCAGCCGCTCACCAGCGCTGGGCCCGCGC TTTCCCACCTGCCTCCACTCTGGGGGCTGGC ACACGCTGCGTCCGGGCA	CTGGCCGG ACGCCCAT CTtctggCCC GGAGTGAT GCAGAGCC
INS left homology arm	GCCGCCCCCAGCCACCCCCTGCTCCTGGCG CTCCCACCCAGCATGGGCAGAAGGGGGCAG GAGGCTGCCACCCAGCAGGGGGTCAGGTGC ACTTTTTTAAAAAGAAGTTCTCTTGGTCACGT CCTAAAAGTGACCAGCTCCCTGTGGCCCAGT CAGAATCTCAGCCTGAGGACGGTGTTGGCTT CGGCAGCCCCGAGATACATCAGAGGGTGGG CACGCTCCTCCCTCCACTCGCCCCTCAAACA AATGCCCCGCAGCCCATTTCTCCACCCTCAT TTGATGACCGCAGATTCAAGTGTTTTGTTAAG TAAAGTCCTGGGTGACCTGGGGTCACAGGGT GCCCCACGCTGCCTGCCTCTGGGCGAACAC CCCATCACGCCCGGAGGAGGGCGTGGCTGC CTGCCTGAGTGGGCCAGACCCCTGTCGCCA GGCCTCACGGCAGCTCCATAGTCAGGAGAT GGGGAAGATGCTGGGGACAGGCCCTGGGGA GAAGTACTGGGATCACCTGTTCAGGCTCCCA	-

	CTGTGACGCTGCCCCGGGGCGGGGAAGGA GGTGGGACATGTGGGCGTTGGGGCCTGTAG GTCCACACCCAGTGTGGGTGACCCTCCCTCT AACCTGGGTCCAGCCCGGCTGGAGATGGGT GGGAGTGCGACCTAGGGCTGGCGGGCAGG CGGGCACTGTGTCTCCCTGACTGTGTCCTCC TGTGTCCCTCTGCCTCGCCGCTGTTCCGGAA CCTGCTCTGCGCGGCACGTCCTGGCAGTGG GGCAGGTGGAGCTGGGCGGGGGCCCTGGT GCAGGCAGCCTGCAGCCCTTGGCCCTGGAG GGGTCCCTGCAGAAGCGTGGCATTGTGGAA CAATGCTGTACCAGCATCTGCTCCCTCTACC AGCTGGAGAACTACTGCAAC	
INS right homology arm	TAGACGCAGCCCGCAGGCAGCCCCACACCC GCCGCCTCCTGCACCGAGAGAGATGGAATAA AGCCCTTGAACCAGCCCTGCTGTGCCGTCTG TGTGTCTTGGGGGCCCTGGGCCAAGCCCCA CTTCCCGGCACTGTTGTGAGCCCCTCCCAGC TCTCTCCACGCTCTCTGGGTGCCCACAGGTG CCAACGCCGGCCAGGCCCAGCATGCAGTGG CTCTCCCCAAAGCGGCCATGCCTGTCGGCTG CCTGCTGCCCCCACCCTGTGGCTCAGGGTC CAGTATGGGAGCTGCGGGGGTCTCTGAGGG GCCAGGGGTGGTGGGGCCACTGAGAAGTGA CTTCTTGTTCAGTAGCTCTGGACTCTTGGAGT CCCCAGAGACCTTGTTCAGGAAAGGGAATGA GAACATTCCAGCAATTTTCCCCCCACCTAGC CCTCCCAGGTTCTATTTTTAGAGTTATTTCTG ATGGAGTCCCTGTGGAGGGAGGAGGCTGGG CTGAGGGAGGGGGTCCTGCAGGGCGGGGG GCTGGGAAGGTGGGGAGAGGCTGCCGAGAG CCACCCGCTATCCCCAGCTCTGGGCAGCCC CGGGACAGTCACACACCCTGGCCTCGCGGC CCAAGCTGGCAGCCGTCTGCAGCCACAGCTT ATGCCAGCCCAGGTCCAGCCAGACACCTGA GGGACCCACTGGTGCCTTGGAGGAAGCAGG AGAGGTCAGATGGCACCATGAGCTGGGGCA GGTGCAGGGACCGTGGCAGCACCTGGCAGG GCCTCAGAACCCATGCCTTGGGCACCCCGG CCATGAGGCCCTGAGGATTGCAGCCCAGGA GAAGCAGGGAACCGCCAGGGCCACAGGGGC AGAGACCAGGGCCAGGGTCCCCCTGCAGCC CCTTAGCCCACCCCCTCCCAGTAAGCAGGGC TGCTTGGCTGGCTTCCTTTGCTACAGACCTG CTGCT	-
OCT4 left homology arm	ACTAGTAACGGCCGCCAGTGTGCTGGAATTC GCCCTTTTCCTGCAGGTCCCACCTGCACAGA TATGCAAAGCAGAAACCCTCGTGCAGGCCCG AAAGAGAAAGCGAACCAGTATCGAGAACCGA GTGAGAGGCAACCTGGAGAATTTGTTCCTGC AGTGCCCGAAACCCACACTGCAGCAGATCAG CCACATCGCCCAGCAGCTTGGGCTCGAGAA	GCTCTCCC ATGCATTC AAAC/tgagg

	GGATGTGAGTGCCATGTCTCTCTGCGGGCTC CATCTCTTTCCCCTGTCACCACCTCGCTTTCC CTAGCTCTGGCTCCTCCAACTGCTCTAGGGC TGTTGGCTTTGGACAGAATGTCCAAGCAGTC AGGCCTGTCTCAGCTCATTCTCTAATGTCCTC CTCTAACTGCTCTAGGGCTGTTGGCTTTGGA TAGAATGTCCAAGCAGAGTCAGGCCCGTCTC AGCTCATTGTCTAATGTCATTCTCCTTTCTGT CATTCACTTGCAGGTGGTCCGAGTGTGGTTC TGTAACCGGCGCCAGAAGGGCAAGCGATCA AGCAGCGACTATGCACAACGAGAGGATTTTG AGGCTGCTGGGTCTCCTTTCTCAGGGGGACC AGTGTCCTTTCCTCTGGCCCCAGGCCCCAT TTTGGTACCCCAGGCTATGGGAGCCCTCACT TCACTGCACTGTACTCCTCGGTCCCTTTCCCT GAGGGGGAAGCCTTTCCCCCTGTCTCCGTCA CCACTCTGGGCTCTCCCATGCATTCAAAC	
OCT4 right homology arm	GGTGCCTGCCCTTCTAGGAATGGGGGACAG GGGGAGGGGAGGAGCTAGGGAAAGAAAACC TGGAGTTTGTGCCAGGGTTTTTGGGATTAAG TTCTTCATTCACTAAGGAAGGAATTGGGAACA CAAAGGGTGGGGGCAGGGGAGTTTGGGGCA ACTGGTTGGAGGGAAGGTGAAGTTCAATGAT GCTCTTGATTTTAATCCCACATCATGTATCAC TTTTTTCTTAAATAAAGAAGCCTGGGACACAG TAGATAGACACACTTATCTTGGTTTGTCCTTC AGTTACTGAGGTAGGGATGGGAATATCCAAT GCTCATACCCAAGTGACCCTGAAACTAAGGT GCCATTTACACTCCTTAAGGTCACACAACATC AGAGGGAGAGCTGGGATTGCAGCCAAGTTTA TTTGTACAGGGCCCTGTGATAGGCTAGTTCC CAAAAGCCTGTGATGCAAGAACTTTTGCCCA TAGACTCAGTCACCATGTAGCTGTTACCTGTT CAGAGCTGGCTTTTTGCTTTCCCACCCTACTC TGGAATTCTTAAATGGCTTTATACTTAGAAAT CATCTTATTTCTGTTGAACCTAGATCACCCCA ACCAGAAACTTCTATTAATACTTTGTGCTTTCT TGATACCAGGGTCTATTTGGTTTCCACTTAAG GTTTTTGCATACTCTGCCCATAAGTGACTCAT TAGTTACGGCCGGCCAAAAGGGCGAATTCTG CAGATATCCATCACACTG	gctctcccatgc attcaaactga GG

Supplementary Table 7: Homology arm sequences for donor integration. Guide sequences are mutated or interrupted by the donor cassette to prevent binding to homologous donors after integration. Mutated sequences and interrupted sequences are shown in lower case.

Gene target	Primer	Region of amplification	Size	Sequence
WNT5A	F	5’		GCAGAAGTATTGGGGCTTGA
WNT5A	R	5’		GCTTGGCTGGACGTAAACTC
WNT5A	F	3’		ATTGCATCGCATTGTCTGAG

WNT5A	R	3’		CAAAAGCAGAGGCAAACAAA
NKX6.1	F	Cut site		CACGCTTTAGCAGCCTGAG
NKX6.1	R	Cut site		CTAACTGGTGTGATTCCGGC
INS	R	3’		TTGCTACAGACCTGCTGCTC
OCT4	F	Cut site		GTACTCCTCGGTCCCTTTCC
OCT4	R	Cut site		CAAAAACCCTGGCACAAACT
eCFP	F	Cloning		CTAGGGAATTAATTCACAGCCACC
eCFP	R	Cloning		CGCGTAGAGTCGCGGTGATCTAGA

Supplementary Table 8: List of primers used for NHEJ and integration assays. (eCFP: enhanced Cerulean fluorescent protein).

Gene	Forward primer	Reverse primer
OCT3/4//POU5F1	tgggctcgagaaggatgtg	gcatagtcgctgcttgatcg
NANOG	ttgggactggtggaagaatc	gatttgtgggcctgaagaaa
REX1/ZFP42	aggatctcccacctttccaa	caggtagcacacctcctgc
LIN8	acccttccatgtgcagctta	tgtaagtggttcaacgtgcg
SOX2	agaaaaacgagggaaatggg	gtcatttgctgtgggtgatg
TERT	atcagccagtgcaggaactt	agctgacgtggaagatgagc
FOXA2/HNF3B	gctactcctccgtgagcaac	gggctcatggagttcatgtt
SOX17	ggcgcagcagaatccaga	ccacgacttgcccagcat
GATA4	caggcgttgcacagatagtg	cccgacaccccaatctc
HNF4A	catagcttgaccttcgagtgc	cgtggtggacaaagacaaga
CXCR4	caccgcatctggagaacca	gcccatttcctcggtgtagtt
BRA/TBX1	gctgaagtgcatccctgc	agcgaggaggaagggaac
GATA2	gccataaggtggtggttgtg	cacaagatgaatgggcagaa
RUNX1	caatggatcccaggtattgg	ccactccactgcctttaacc
HAND1	aatcctcttctcgactgggc	ccttcaaggctgaactcaaga
BRCA1	gaaggccctttcttctggtt	agagtgtcccatctgtctgga
BRCA2	acaaatagacgaaaggggca	gccccttcacttcagcaaat
TUBB3	agtcgcccacgtagttgc	cgcccagtatgaggagat
NESTIN	tctttgctcccagtcctgag	gggctctgatctctgcatct
PAX6	tgtgtgctctgaaggtcagg	cctggagctctgtttggaag
RAD51	ctggtggtctgtgttgaacg	ctgagggtacctttaggcca
RAD52	gctgtcacgtcctccaaga	cttccccttgtccatagcct
XRCC4	tggactgggacagtttctga	tcagttcaccaacatatttccc
XRCC5	tgaggaagcgagtaaccagctcataaat cacatc	atgcagtctatgctcttcataaaata cg
XRCC6	agcactcagcaggttaaagctgaagctc aac	gattataaatgcccacagagatca ctat
LIG4	agcttgcccgaggccagttaaacga	aaaaggaacgtgagatgcaaca
TBP	gttctgaataggctgtgggg	acaacagcctgccaccttac
ACTB	ccttgcacatgccggag	gcacagagcctcgcctt
18s rRNA	tcggaactgaggccatgatt	ctttcgctctggtccgtctt

Supplementary Table 9: List of primers used for qRT-PCR shown in Figure 5 and Supplementary Figure 5.

Antigen	Supplier, species, catalog number	Dilution
OCT3/4	SCBT, mouse, sc-5279	1:100
SOX2	SCBT, goat, sc-17320	1:100

TRA1-81	Abcam, mouse, 16289	1:200
SSEA-4	DSHB, mouse, MC-813-70	1:50
SOX17	R&D Systems, goat, AF1924	1:500
FOXA2/HNF3B	Millipore, rabbit, 07-633	1:250
PDX1	R&D Systems, goat, AF2419	1:500
CTNT	Abcam, rabbit, ab45932	1:500
NKX2.5	DSHB, mouse, PCRP-NKX2-5-3B4	1:100

Supplementary Table 10: List of antibodies and dilutions used in Figure 5.

Gene name	#	Category	H1 UT	H1 DMSO	H1 Noco	H1 ABT	HUES8 UT	HUES8 DMSO	HUES8 Noco	HUES8 ABT	DE	CP	NP
OCT4	1	pluripotency	10.2	11.5	9.8	10.5	13.4	12	10.1	11			
	2	pluripotency	10.64	11.68	9.9	10.1	14.15	12.5	10.3	10.95			
	3	pluripotency	10.04	11.2	9.23	9.89	13.89	11.67	10.44	12.05			
NANOG	1	pluripotency	7.3	5.6	5.85	6.2	9.5	7.1	7.45	8.8			
	2	pluripotency	6.8	5	5.57	6.2	9.15	7.93	8.22	8.73			
	3	pluripotency	6.56	4.8	5.72	6.89	8.66	7.44	7.72	8.16			
REX1	1	pluripotency	4.1	4.56	3.99	4.53	4.6	4.4	4.73	4.21			
	2	pluripotency	4.22	4.67	4.13	4.38	4.17	4.84	5.01	4.44			
	3	pluripotency	4.78	4.8	4.42	5.01	4.06	4.25	4.66	4.92			
GDF3	1	pluripotency	7.28	7.8	7.94	7.1	6.54	6.32	7.04	6.22			
	2	pluripotency	6.9	8.12	7.28	6.92	6.93	7.93	8.11	8.73			
	3	pluripotency	6.67	7.74	7.92	6.19	7.05	7.44	7.72	8.16			
LIN28	1	pluripotency	9.3	9.6	8.85	10.2	9.5	9.1	8.45	8.8			
	2	pluripotency	9.8	9	8.97	11.1	9.15	8.93	8.11	8.73			
	3	pluripotency	8.56	8.4	7.52	8.89	8.66	8.77	9.72	8.16			
FOXA2	1	endoderm	1.23	1.5	1.2	1.74	1.45	1.67	1.74	1.85	159.1		
	2	endoderm	1.11	1.22	1.05	1.64	1.43	1.84	1.6	1.65	147		
	3	endoderm	1.37	1.56	1.15	1.8	1.69	1.62	1.63	1.48	161		
GATA4	1	endoderm	1.6	1.32	1.3	1.1	1.1	0.96	0.76	1.03	53.8		
	2	endoderm	1.41	1.25	1.45	1.02	0.98	0.88	0.64	1.14	58.1		
	3	endoderm	1.66	1.28	1.27	1.16	1.24	1.19	0.75	0.99	56.7		
CXCR4	1	endoderm	2.4	3	2.68	2.11	1.43	1.77	1.94	1.88	66.1		
SOX17	1	endoderm	1.8	2.5	2.89	1.5	2.4	2.71	2.89	2.16	127.8		
	2	endoderm	1.96	2.08	2.89	1.75	2.2	2.65	2.51	2.37	144.3		
	3	endoderm	2.08	2.89	2.41	1.88	2.11	2.43	2.18	2.02	125		
BRA(T)	1	mesoderm	1.96	2.28	2.14	2.35	1.67	1.99	2.12	2.11		43	
	2	mesoderm	1.85	2.49	2.15	2.22	1.59	2.11	2.36	2.18		48.11	
	3	mesoderm	2.15	2.51	1.99	2.21	1.42	1.94	2.56	2.74		51.95	
TPNT	1	mesoderm	1.45	1.85	1.77	2.19	2.05	1.78	1.81	1.99		85.64	
	2	mesoderm	1.84	2.00	2.14	1.68	1.93	2.39	2.3	1.73		81.5	
	3	mesoderm	1.42	1.67	1.94	1.99	1.72	2.19	2.3	2.16		81.44	
NKX2.5	1	mesoderm	0.78	1.29	1.11	1.21	0.93	1.25	0.72	1.33		93.6	
	2	mesoderm	1.15	1.37	1.25	1.34	1.17	1.03	0.69	1.19		99.7	
	3	mesoderm	1.02	1.56	1.73	0.69	0.97	0.85	1.13	1.41		98.1	
PAX6	1	ectoderm	2.11	2.50	2.74	2.91	2.60	3.15	2.74	3.01			171.9
	2	ectoderm	2.84	3.07	3.11	2.67	3.50	2.93	2.93	2.45			168.5
	3	ectoderm	2.88	3.67	3.21	3.00	3.24	2.86	2.61	2.88			170.2
SOX1	1	ectoderm	1.67	1.84	1.93	1.52	1.67	1.89	2.15	2.28			72.4
	2	ectoderm	2.51	1.11	1.78	1.46	2.02	2.06	1.83	2.51			72.1
	3	ectoderm	2.55	1.44	1.72	1.62	2.04	2.23	2.33	1.85			67,8

Supplementary Table 11: Synchronized cells have similar gene expression patterns to unsynchronized cells. qPCR values shown for three replicates.
